# Taming chlorophylls by early eukaryotes underpinned algal interactions and the diversification of the eukaryotes on the oxygenated Earth

**DOI:** 10.1038/s41396-019-0377-0

**Published:** 2019-02-26

**Authors:** Yuichiro Kashiyama, Akiko Yokoyama, Takashi Shiratori, Sebastian Hess, Fabrice Not, Charles Bachy, Andres Gutierrez-Rodriguez, Jun Kawahara, Toshinobu Suzaki, Masami Nakazawa, Takahiro Ishikawa, Moe Maruyama, Mengyun Wang, Man Chen, Yingchun Gong, Kensuke Seto, Maiko Kagami, Yoko Hamamoto, Daiske Honda, Takahiro Umetani, Akira Shihongi, Motoki Kayama, Toshiki Matsuda, Junya Taira, Akinori Yabuki, Masashi Tsuchiya, Yoshihisa Hirakawa, Akane Kawaguchi, Mami Nomura, Atsushi Nakamura, Noriaki Namba, Mitsufumi Matsumoto, Tsuyoshi Tanaka, Tomoko Yoshino, Rina Higuchi, Akihiro Yamamoto, Tadanobu Maruyama, Aika Yamaguchi, Akihiro Uzuka, Shinya Miyagishima, Goro Tanifuji, Masanobu Kawachi, Yusuke Kinoshita, Hitoshi Tamiaki

**Affiliations:** 1 Graduate School of Engineering, Fukui University of Technology, Fukui, Fukui, Japan; 2 Department of Environmental and Biological Chemistry, Faculty of Engineering, Fukui University of Technology, Fukui, Fukui, Japan; 3 Graduate School of Life Sciences, Ritsumeikan University, Kusatsu, Shiga, Japan; 4 Center for Environmental Biology and Ecosystem Studies, National Institute for Environmental Studies, Tsukuba, Ibaraki, Japan; 5 Faculty of Life and Environmental Sciences, University of Tsukuba, Tsukuba, Ibaraki, Japan; 6 Center for Regional Environmental Research, National Institute for Environmental Studies, Tsukuba, Ibaraki, Japan; 7 Department of Marine Biodiversity Research, Japan Agency for Marine–Earth Science and Technology, Yokosuka, Kanagawa, Japan; 8 Life Sciences Centre, Dalhousie University, Halifax, Nova Scotia Canada; 9 Sorbonne University, CNRS, UMR7144, Adaptation and Diversity in Marine Environment (AD2M) laboratory, Ecology of Marine Plankton team, Station Biologique de Roscoff, Place Georges Teissier, 29680 Roscoff, France; 10 National Institute of Water and Atmospheric Research, 301 Evans Bay Parade, Wellington, 6021 New Zealand; 11 Graduate School of Science, Kobe University, Kobe, Hyogo, Japan; 12 Division of Applied Life Sciences, Graduate School of Life and Environmental Sciences, Osaka Prefecture University, Sakai, Osaka, Japan; 13 Department of Life Science and Biotechnology, Faculty of Life and Environmental Science, Shimane University, Matsue, Shimane, Japan; 14 Institute of Hydrobiology, Chinese Academy of Sciences, Wuchang District, Wuhan, China; 15 Department of Environmental Science, Faculty of Science, Toho University, Funabashi, Chiba, Japan; 16 Graduate School of Environment and Information Sciences, Yokohama National University, Yokohama, Kanagawa, Japan; 17 Graduate School of Natural Science, Konan University, Kobe, Hyogo, Japan; 18 Institute for Integrative Neurobiology, Konan University, Kobe, Hyogo, Japan; 19 Faculty of Science and Engineering, Konan University, Kobe, Hyogo, Japan; 20 Graduate School of Life and Environmental Sciences, University of Tsukuba, Tsukuba, Ibaraki, Japan; 21 Graduate School of Science, Kyoto University, Kyoto, Kyoto, Japan; 22 Biotechnology Laboratory, Electric Power Development Co., Ltd., Kitakyusyu, Fukuoka Japan; 23 Institute of Engineering, Tokyo University of Agriculture and Technology, Koganei, Tokyo, Japan; 24 Kobe University Research Center for Inland Seas, Hyogo, Kobe, Japan; 25 Department of Cell Genetics, National Institute of Genetics, Mishima, Shizuoka, Japan; 26 National Museum of Nature and Science, Tsukuba, Ibaraki, Japan

**Keywords:** Microbial ecology, Cellular microbiology, Biochemistry, Biogeochemistry

## Abstract

Extant eukaryote ecology is primarily sustained by oxygenic photosynthesis, in which chlorophylls play essential roles. The exceptional photosensitivity of chlorophylls allows them to harvest solar energy for photosynthesis, but on the other hand, they also generate cytotoxic reactive oxygen species. A risk of such phototoxicity of the chlorophyll must become particularly prominent upon dynamic cellular interactions that potentially disrupt the mechanisms that are designed to quench photoexcited chlorophylls in the phototrophic cells. Extensive examination of a wide variety of phagotrophic, parasitic, and phototrophic microeukaryotes demonstrates that a catabolic process that converts chlorophylls into nonphotosensitive 13^2^,17^3^-cyclopheophorbide enols (CPEs) is phylogenetically ubiquitous among extant eukaryotes. The accumulation of CPEs is identified in phagotrophic algivores belonging to virtually all major eukaryotic assemblages with the exception of Archaeplastida, in which no algivorous species have been reported. In addition, accumulation of CPEs is revealed to be common among phototrophic microeukaryotes (i.e., microalgae) along with dismantling of their secondary chloroplasts. Thus, we infer that CPE-accumulating chlorophyll catabolism (CACC) primarily evolved among algivorous microeukaryotes to detoxify chlorophylls in an early stage of their evolution. Subsequently, it also underpinned photosynthetic endosymbiosis by securing close interactions with photosynthetic machinery containing abundant chlorophylls, which led to the acquisition of secondary chloroplasts. Our results strongly suggest that CACC, which allowed the consumption of oxygenic primary producers, ultimately permitted the successful radiation of the eukaryotes throughout and after the late Proterozoic global oxygenation.

## Introduction

The partial pressure of molecular oxygen (*p*O_2_) in Earth’s atmosphere is thought to have increased rapidly at the end of the last “Snowball Earth” event (the Marinoan glaciation, which ended 635 million years ago) [1–3]. This would have dramatically modified the biochemical constitutions of organisms and selected the ancestral lineages of extant life from the preexisting diversity. Mitochrondria, as respiratory machinery, were probably present in the cells of the last eukaryotic common ancestor (LECA) [4, 5], and undoubtedly powered the successful radiation of its descendants in the oxygenated world. However, the elevated *p*O_2_ conditions of the modern world are a major potential source of oxidative stress (if not death) to organisms. Moreover, molecular oxygen is even destructive when it is photosensitized by “phototoxic” biomolecules, such as chlorophylls [6].

Yet, as the central pigment in photosynthesis, chlorophyll is an essential factor in the modern biosphere. The overwhelming majority of energy used in the modern biosphere is derived from the photoexcitation of light-harvesting chlorophylls, which must be perfectly oriented in photosynthetic proteins to correctly utilize and convert the energy of captured photons to organic matter [7]. However, chlorophyll is a mixed blessing for living organisms: excited chlorophylls photosensitize molecular oxygen, thereby generating reactive oxygen species, such as singlet oxygen. Singlet oxygen is particularly cytotoxic, so chlorophylls can also be considered phototoxic. Consequently, the management of that phototoxicity must have been a central issue in the chlorophyll-dependent biosphere on Earth.

Althoughx our knowledge of how living organisms have managed phototoxins is still limited, a biochemical strategy used to combat the phototoxicity of chlorophylls has recently been reported in algivorous microeukaryotes (i.e., unicellular eukaryotes that use phagocytosis to feed on algae). Within the phagosomes of microeukaryotes, the algal chlorophylls are rapidly catabolized to 13^2^,17^3^-cyclopheophorbide enols (CPEs) [8], which are neither fluorescent nor photosensitive (Supplementary Fig. S1). In this way, algivorous microeukaryotes effectively detoxify their algal prey [8–10]. Kashiyama et al. [8] detected CPEs in virtually all aquatic environmental samples tested, including pond and lake water, coastal and pelagic marine water, and freshwater and marine sediments. The ubiquity of CPEs suggests that CPE-accumulating chlorophyll catabolism (CACC) contributes greatly to the turnover of chlorophylls in aquatic environments, in which ~50% of global primary production takes place [11]. However, accumulation of CPEs has to date been reported in cultures including only single strains representing each of four major eukaryotic assemblages (MEAs): Rhizaria, Alveolata, Stramenopiles, and Haptista [8, 12], along with enigmatic occurrences in several strains of dinoflagellates [13, 14]. The distribution of the potential for CACC and the role of this process in aquatic chlorophyll degradation in certain organismal groups are still largely unknown. Here, we demonstrate that CACC is phylogenetically ubiquitous in extant eukaryotes.

## Materials and methods

### Preparation of authentic samples and standard solutions for HPLC analysis

Authentic samples of chlorophyll derivatives used for the identification of compounds in the high-performance liquid chromatography (HPLC) analysis were prepared using previously described methods [8–10], including chlorophylls *a*/*b* (Chls-*a*/*b*), pheophytins *a*/*b* (Phes-*a*/*b*), pyropheophytins *a*/*b* (pPhes-*a*/*b*), pheophorbide *a* (PPB-*a*), pyropheophorbide *a* (pPPB-*a*), 13^2^,17^3^-cyclopheophorbides *a*/*b* enol (cPPBs-*a*E/*b*E), and (13^2^*R*)- and (13^2^*S*)-hydroxychlorophyllones *a* ([R/S]-hCPLs-*a*). Because cPPB-*a*E is stabilized in deoxygenated anisole [8], all authentic standard solutions used in this study were prepared in anisole (ReagentPlus^®^ grade, Sigma-Aldrich, St. Louis, USA). Molar concentrations were spectrographically determined with reference to the previously reported molar extinction coefficients of the Chl-*a* derivatives [8] using a Hitachi U-3500 spectrophotometer (Hitachi, Ltd., Tokyo, Japan).

### HPLC analysis

Analytical HPLC was performed with a Shimadzu Nexera X2 liquid chromatography system, comprising a CBM-20A communication bus module, two DGU-20A3R/5R HPLC degassing units, three LC-30AD solvent delivery units constituting a ternary pumping system, an SIL-30AC autosampler, a CTO-20AC column oven, and an SPD-M30A photodiode array (PDA) detector with a high-sensitivity capillary flow cell (optical path: 85 mm; Shimadzu, Kyoto, Japan). The system was coupled to a personal computer configured to run the Shimadzu LabSolution software. Reverse-phase HPLC was performed under the following conditions: column, Zorbax Eclipse Plus C18 (Rapid Resolution HT, 4.6 × 30 mm, 1.8 μm silica particle size; Agilent Technologies, Santa Clara, USA); eluent, the ternary gradient program summarized in Supplementary Table S1; flow rate, 1.00 mL min^−1^; range of wavelengths detected with PDA, 300–700 nm. All the mobile phases were degassed in vacuo with ultrasonication and sealed under argon. The mobile-phase reservoir bottles were designed to prevent any contact between the mobile phases and air during analysis. All solvents used for the analytical HPLC mobile phases were HPLC grade, and were purchased from Nacalai Tesque (Kyoto, Japan).

### High-resolution mass spectrometry analysis

High-resolution mass spectra were recorded on a Bruker micrOTOF II spectrometer (Billerica, USA), connected to an HPLC system via an atmospheric pressure chemical ionization (APCI) interface. The HPLC system consisted of a CBM-20A communication bus module, a DGU-20A3R HPLC degassing unit, and an LC-20AD solvent delivery unit. An isolated fraction containing compound-X_*a*, obtained with the analytical HPLC system described above, was subjected to an high-resolution mass spectrometry (HRMS) analysis; methanol was used as the mobile phase for HPLC and was introduced at the APCI interface.

### Microeukaryote culture experiments

One-hundred and eighty-three strains were cultured and tested for their ability to produce CPEs and degrade chlorophylls (Supplementary Table S2). Of the 73 phagoheterotrophic or phagomixotrophic microeukaryote strains examined in this study, 32 were cultured with a known algal prey (either suggested by the culture collection and/or reported in previous studies); 15 (including the 12 strains originally isolated) were cultured in de novo combinations with eukaryotic dietary algae; and 26 strains (including 9 strains originally isolated) that had been maintained with bacterial diets and/or are generally regarded as bacterivores, were co-cultured with picocyanobacteria, and predation on the cyanobacterial cells was confirmed with microscopy. Four organisms that contained either endosymbiotic algae or kleptochloroplasts were also isolated from natural environments for pigment analysis. Finally, 112 strains of algae (including 6 originally isolated strains) were cultured under the photoautotrophic conditions, which included in the algal diets of the phagoheterotrophic microeukaryotes examined; they were grown with their common culture conditions until late stages of the stationary phase or early stages of the death phase. They were then sampled for pigment analysis.

### Organelle fractionation by density gradient ultracentrifugation

Cells of *Euglena gracilis* strain-z grown in a photoautotrophic culture (Cramer–Myers medium) [15] in a late stationary phase of growth were harvested by centrifugation, washed once and resuspended in fresh Cramer–Myers medium. They were then disrupted with the BioNeb disruption system (Glas-Col, LLC, IN) in which the lysate was immediately poured into sorbitol buffer (final concentration: 1 M sorbitol, 50 mM HEPES, 2 mM Na_2_-EDTA, 1 mM MnCl_2_, 0.2 mM MgCl_2_, 1 mM Na_4_P_2_O_7_, 5 mM isoascorbic acid, 5 mM glutathione, pH 6.8). The lysate was then loaded onto a discontinuous sorbitol density gradient (2/2.5/2.75/3 M sorbitol in a buffer containing 50 mM HEPES, 2 mM Na_2_-EDTA, 1 mM MnCl_2_, 0.2 mM MgCl_2_, 1 mM Na_4_P_2_O_7_, 5 mM isoascorbic acid, 5 mM glutathione, pH 6.8 space left in the tube over the loaded sample filled with 0.33 M solbitol-buffer) and at 4 °C and subjected to ultracentrifugation at 100,000 × *g* on a S52ST rotor (Hitachi-Koki, Japan) for 60 min at 4 °C. A portion of the isolated fractions was immediately microscopically observed. The rest of fraction was resuspended in 1 M sorbitol buffer and pelleted with high-speed centrifugation for further analysis.

### Sample preparation and pigment extraction for HPLC analysis

The whole contents of experimental cultures were gently suspended and aliquoted into 1.5 mL polypropylene microtubes. The suspended matter in the samples was pelleted with high-speed centrifugation, and the supernatants were completely removed. The pelleted samples were instantly frozen with liquid nitrogen. When the pigments were extracted for HPLC analysis, aliquots of acetone were added to the frozen microtubes on a tube rack chilled below 0 °C and then placed in an ice-cooled ultrasonication bath for extraction. After the samples were ultrasonically homogenized for a few minutes, the acetone supernatants were immediately separated from particulate material by centrifugation and directly injected into the HPLC apparatus for analysis, with no further pretreatment, such as filtration. All these extraction steps were performed under an argon atmosphere in a glove box, to prevent the samples and extracts contacting oxygen.

## Results

**Fig. 1:**
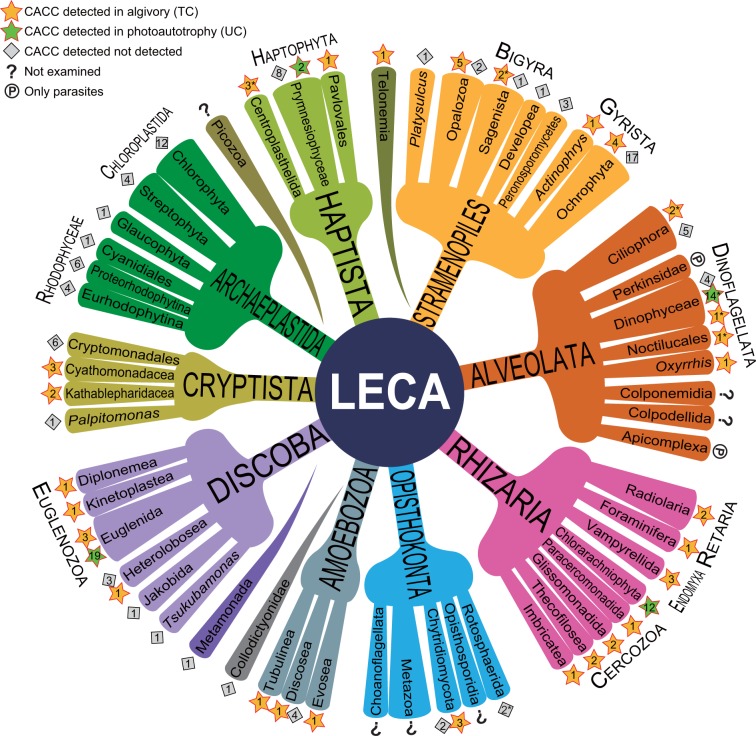
Scheme of eukaryote evolution and classification showing major eukaryotic assemblages (MEAs) displaying CPE-accumulating chlorophyll catabolism. Stars indicate accumulation of CPEs detected: yellow stars, data from two-membered co-cultures (algivory); green stars, data from unialgal cultures (chloroplast dismantling). Diamonds indicate no accumulation of CPE detected. Number in each star or diamond denotes the total number of species examined in the present study and/or previously reported (listed in Supplementary Table S2). Numbers with asterisks are data from previous reports [8, 12–14]. Among the nine MEAs, Archaeplastida examined in the present study consists exclusively of phototrophs, whereas Amoebozoa and Opisthokonta consist exclusively of heterotrophs. Each of the other six supergroups includes both phototrophs and algivores; thus, we examined both phototrophic and algivorous cultures across all the six MEAs

**Fig. 2:**
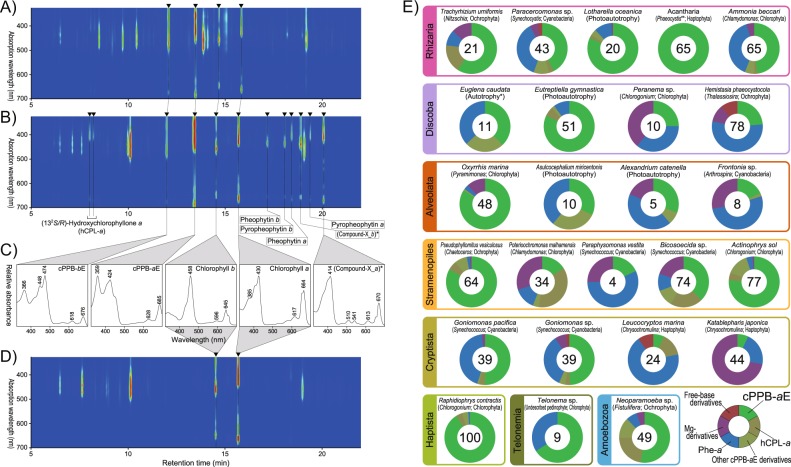
Identification and quantitative illustration of CPEs and other chlorophyll derivatives. Left: Three-dimensional (3D) HPLC chromatograms of extracts of **a** an aged unialgal culture of a euglenophyte (*Eutreptiella* sp. CCMP389); and **b** a two-membered co-culture of a algivorous stramenopile (*Actinophris sol*) fed a dietary green alga (*Chlorogonium capillatum*); **c** HPLC online visible absorption spectra of major chlorophylls and their derivatives; **d** 3D HPLC chromatogram of an extract of *C. capillatum* only (an unialgal culture). Right: **e** Donut charts showing the relative abundances of the derivatives of chlorophyll *a* (Chl-*a*) in representative cultures in which CPEs were detected. These included 13^2^,17^3^-cyclopheophorbide *a* enol (cPPB-*a*E) and other miscellaneous derivatives: (13^2^*R*/*S*)-hydroxychlorophyllone *a* (hCPL-*a*), other cPPB-*a*E derivatives (pyropheophytin *a* and compound-X_*a*; Supplementary Fig. S1), pheophytin *a* (Phe-*a*), Mg-chelated derivatives of Chl-*a* (Chl-*a* allomers and chlorophyllide *a*), and free-base derivatives of Chl-*a* (pheophorbide *a* and pyropheophorbide *a*). Species names in parentheses indicate the dietary algae in two-membered co-cultures. Numerals in each doughnut chart indicate the ratio of the plotted derivative to the total Chl-*a* derivatives (the plotted derivatives plus intact Chl-*a*) in each analysis as a percentage. Therefore, 100 indicates the complete alteration of the originally produced Chl-*a*

**Fig. 3:**
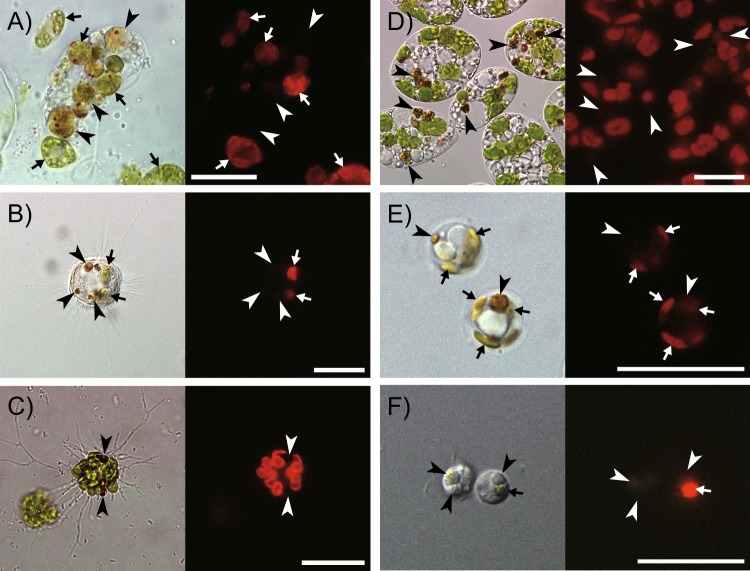
Microscopic documentation of the degradation of chloroplasts. Differential interference (left) and fluorescent images (excitation: 400–440 nm) (right). **a** Cell of heterotrophic euglenid *Peranema trichophorum* (center) containing phagosomes of variable color, which is surrounded by live cells of the green alga *Chlorogonium capillatum* (arrows). Among the ingesta, the autofluorescence intensity differs more than the color, indicating the progress of chlorophyll catabolism together with chloroplast digestion (arrowheads); **b** cell of the heterotrophic heliozoan (Haptista) *Choanocystis* sp. that had ingested *C. capillatum*, demonstrating that chlorophyll autofluorescence gradually disappeared in an early stage of digestion (arrowheads); **c** cells of *Euglena gracilis* in an aged culture, showing the formation of brown granules after chloroplast dismantling, where the loss of chlorophyll autofluorescence was observed in the earliest stage. Cells of **d** the chlorarachniophyte *Chlorarachnion reptans*; and **e** the haptophyte *Calyptrosphaera sphaeroidea*, respectively, both showing the formation of nonfluorescent reddish-brown granules (arrowheads); **f** cells of *Palpitomonas bilix* that had ingested a pedinophycean green alga, demonstrating that the pigmentation of the dietary alga was also lost within the phagosome (arrow: in the earliest stage of digestion; arrowheads: in the later stages), in contrast to the CPE-producing algivores

We examined CACC of heterotrophic microeukaryotes by analyzing the pigments produced in 73 laboratory cultures of diverse algivorous microeukaryotes feeding on microalgae (two-membered co-cultures; TCs) and in three colonies isolated from the environment, each containing a specific microeukaryote with an endosymbiotic alga. The formation of CPEs was observed in a wide diversity of heterotrophic microeukaryotes belonging to eight of the nine accepted MEAs: Rhizaria, Alveolata, Stramenopiles, Haptista, Cryptista, Discoba, Amoebozoa, and Opisthokonta (Fig. 1, Fig. 2, and Supplementary Table S2). A reduction in the chlorophyll autofluorescence of the phagocytosed algae was commonly observed microscopically in the algivores (Fig. 3a, b), suggesting the conversion of chlorophylls into nonfluorescent CPEs within their phagosomes [8].

We also conducted experiments on 112 unialgal cultures (UCs), and accumulation of CPE was found to be common among algae containing secondary chloroplasts, including chlorarachniophytes, phototrophic dinoflagellates (dinophytes) [12–14], haptophytes, and phototrophic euglenids (euglenophytes; Fig. 1, Fig. 2, and Supplementary Table S2). Strikingly, no CPEs were detected in any of the primary photosynthetic organisms tested (i.e., members of the Archaeplastida). Here, we summarize the results on occurrence and distribution of CACC of each MEA with a particular focus on association of algivorous and photosynthetic lineages.

### Rhizaria

All rhizarian species examined displayed the potential of CACC, including both algivorous and photosynthetic species (Figs. 1 and 2, Supplementary Fig. S2A, and Supplementary Table S2). We examined nine lineages of Cercozoa and Endomyxa, two of the three subgroups of Rhizaria [16], each of which contains algivorous species. CPEs were the primary derivatives of chlorophylls in all TCs with cercozoans and endomyxans (Fig. 2e), as shown in *Abollifer globosa* in previous reports [2, 17]. Besides well-known endomyxan algivores such as vampyrellid amoebae and viridiraptorid amoeboflagellates [18–20], this also included experimentally established TCs of a cercozoan *Paracercomonas* sp. [10] with the cyanobacterium *Synechococcus leopoliensis*. Although *Paracercomonas* is generally known to feed on heterotrophic bacteria [21], we detected accumulation of CPEs in our experiments with *S. leopoliensis*. This indicates that the bacterivorous *Paracercomonas* retained the metabolic capacity to detoxify chlorophylls and thus apparently to feed on algae. Many examples of such facultative cyanobacterivory were observed in experimental cultures of other microeukaryotes examined in the present study. The term “Cyano-TCs” is used for such cultures hereafter as well as in Supplementary Table 2.

Significantly, accumulation of CPEs was also identified in all the UCs of chlorarachniophytes, phototrophic cercozoans that possess secondary chloroplasts derived from a green alga [22]. The chlorarachniophytes in aged cultures typically formed brownish–orange globules within their cells (Fig. 3c), when accumulation of CPEs became prominent.

Among three distinct lineages of Retaria, the third subgroup of Rhizaria [16], the heterotrophic foraminifer *Ammonia* sp. exhibited CACC in its TCs when it was fed with the diatom *Fistulifera solaris* (Fig. 2e) and with the green alga *Pyramimonas parkeae*. Chlorophyll-*a*-derived cPPB-*a*E was also produced by two distinct radiolarians harboring algal endosymbionts: a member of the Collodaria with a dinophyte and a member of the Acantharia with a haptophyte, *Phaeocystis* sp. (Fig. 2e). Although cPPB-*a*E was detected in the Collodaria, we cannot exclude the possibility that it was derived from the endosymbiotic dinophyte [13]. On the other hand, the production of cPPB-*a*E in the Acantharia colony is probably attributable to the host cell, since CPE accumulation was not detected in a free-living *Phaeocystis* strain and, generally, seems to be rare among haptophytes (Supplementary Table S2).

### Discoba

CACC was also generally observed among euglenozoan microeukaryotes (euglenids, diplonemids, and kinetoplastids; Fig. 1, Fig. 2 and Supplementary Fig. S2B), including phototrophic euglenids (euglenophytes). Euglenozoa is a major branch of the Discoba, which possibly represent the most basal branch of eukaryotes [23]. It contains microeukaryotes with diverse modes of nutrition, including phagotrophy, osmotrophy, and phototrophy by secondary chloroplasts, derived from a green alga [24]. All the algivorous euglenozoans we examined clearly displayed CACC during the digestion of algae (Figs. 2e and 3a). Importantly, all the euglenophytes in UCs also produced CPEs (Figs. 1, 2a, and c), and similar to chlorarachniophytes, progressively accumulated CPEs after the stationary phase of growth (Supplementary Fig. S3).

We commonly observed reddish-brown to dark-brown globules [25] within the cells of euglenophytes in aged cultures (Fig. 3d), and demonstrated in *Euglena gracilis* that the globules were formed during the dismantling of the chloroplasts, which is apparently associated with CACC (Supplementary Figs. S3–S5). In both photomixotrophic and photoautotrophic cultures of *E. gracilis* strain Z, chlorophyll-*a*-derived cPPB-*a*E and its related compounds (metabolic intermediate compound-X_*a* and a byproduct pPhe-*a*; see Supplementary Fig. S1) gradually accumulated after the stationary phases and finally dominated in the extracts by about 22% and 14% of total chlorophyll derivatives, after about 18 and 153 days of culture, respectively (Supplementary Fig. S3). In such cultures, the cells typically exhibited dismantling of chloroplasts, a process that evidently led to formation of the brown globules. At first, chlorophyll autofluorescence disappeared in the pale chloroplasts, representing the earliest stage of the dismantling. This was followed by shrinking in size and darkening in color (Supplementary Fig. S4A). Transmission electron microscopic images of the shrinking structures typically show formation of lipid bodies surrounded by bundles of membranes that are probable remnant structures of degraded thylakoids (Supplementary Fig. S4B and C). The brown globules isolated by density gradient fractionation exhibited a quantitative concentration of cPPB-*a*E and related compounds (Supplementary Fig. S5A), whereas proteins including photosynthetic components as well as intact chlorophylls were nearly absent (Supplementary Fig. S5B and C), suggesting CACC during the chloroplast dismantling.

### Alveolata, Haptista, Stramenopiles, and Cryptista

Each of these MEAs includes phototrophic groups, namely dinophytes, haptophytes, ochrophytes, and cryptophytes (phototrophic cryptomonads), respectively. Their secondary and tertiary chloroplasts derived from a red alga were acquired independently in each clade [26]. We and previous works [13, 14] identified accumulation of cPPB-*a*E, a chlorophyll-*a*-derived CPE, in UCs of some of these phototrophs.

The production of cPPB-*a*E, chlorophyll-*a*-derived CPE, by dinophytes in UCs was reported previously [13, 14]; here, accumulation of the CPE was detected in UCs of four out of six phototrophic strains (Supplementary Table S2). It is noteworthy that the production of CPEs has associated with the digestion of algae in an algivorous dinoflagellate *Amphidinium* sp. [12], which implies that CACC in dinophytes is also related to the digestion of degraded chloroplasts. This seems to be analogous to the chloroplast dismantling seen in euglenophytes. Here, CACC was detected in the primarily non-photosynthetic algivorous microeukaryote *Oxyrrhis marina* (Fig. 2e and Supplementary Table S2) that branches off the base of dinoflagellates in phylogenies (Supplementary Fig. S2C) [27]. CACC was also previously reported in the dinoflagellate *Noctiluca scintillans*, grown in heterotrophic conditions [12].

Ciliates are also potential CPE producers in aquatic environments because many of them are able to consume microalgae. Goericke et al. [12] reported CPE in the fecal material of *Strombidinopsis acuminatum*. However, CACC seems to occur sporadically among ciliates. From the six species of algivorous and mixotrophic ciliates we tested, only *Frontonia* sp. produced CPEs (Fig. 2e and Supplementary Table S2).

Among the haptophytes, two out of 12 species produced detectable amounts of CPEs (Supplementary Table S2). Formation of brown globules in cells was typically observed in aged cultures of the strains that exhibited CACC (Fig. 3e). Haptophytes are a monophyletic group of algae within the MEA Haptista, hence nested within heterotrophic microeukaryotes [26] (Supplementary Fig. S2D). Importantly, as reported here and in previous studies, CACC is generally present among the Centroplasthelida (centrohelids), a basal group of Haptista representing the heterotrophs (Supplementary Fig. S2D) [9, 21].

An intriguing absence of CACC in UCs was found among ochrophytes and cryptophytes, even if their chloroplasts are derived from a red alga similarly to dinophytes and haptophytes. In TCs, however, many of algivorous Stramenopiles exhibited CACC upon predation of algae, which include some of mixotrophic species of ochrophytes (i.e., chrysophyceans). Furthermore, CPE production was commonly observed among algivorous Cryptista, including three Cyano-TCs of cyathomonadaceans (goniomonads) and two TCs of katablepharids that prey on algae (Fig. 2e and Supplementary Fig. S2E and Supplementary Table S2).

### Amoebozoa and Opisthokonta

All strains we tested in Amoebozoa and Opisthokonta were algivores in TCs because no phototroph has been discovered among these MEAs [16]. Accumulation of CPEs was observed in a half of the Amoebozoa species examined in the present study. CACC was clearly detected in an algivorous strain of *Neoparamoeba* sp. originally identified as a predator of diatoms in nature, in a testate ameba *Arcella* sp. that fed on a diatom species, and a yet undescribed parasitoid amoebozoan belonging to Cutosea that consumes zygnematalean green algae. Among the Opisthokonta species, we found CACC only among the chytrid fungi, when parasitizing diatoms.

## Discussion

The discovery that eukaryotic algae with secondary chloroplasts produce CPEs under photoautotrophic culture conditions extends our understanding of microeukaryotic chlorophyll catabolism beyond algivory. Our microscopic observations suggest that the algal CPE accumulation occurs in association with the dismantling of chloroplasts in parallel with formation of brownish, nonfluorescent globules observed in chlorarachniophytes, euglenophytes, and haptophytes. This indicates a role for the algal CACC in controlled chloroplast degradation. We infer that the CACC observed in each of these four lineages of algae was inherited from phagotrophic ancestors, rather than derived from the green or red algal progenitors of their secondary chloroplasts. For example, because all the algivorous euglenozoan examined clearly displayed accumulation of CPEs during the digestion of algae (Fig. 1 and Supplementary Table S2), the CACC observed in euglenophytes is probably plesiomorphic for the entire Euglenozoa. Because euglenophytes are monophyletic within Euglenozoa [24], algal CACC is most likely have descended from an ancestral euglenozoan host cell. Similarly, we suggest that the CACC of chlorarachniophytes, haptophytes, and dinophytes is also likely descended from their phagotrophic ancestors. In these cases, the metabolic strategy originally used to accommodate the phototoxicity of dietary chlorophylls also allowed for the retention of phagocytosed algae as endosymbionts, and thus facilitated their evolution as secondary chloroplasts. A secure biochemical strategy for the degradation of chlorophylls, such as CACC, must have been essential for microeukaryotes that occasionally dismantled their chloroplasts.

In addition to the CACC associated with chloroplast dismantling, accumulations of CPEs were also observed in some two-membered algal co-cultures, where one alga mixotrophically preys on the other. In particular, a strain of chrysophycean alga *Poterioochromonas malhamensis* exhibited quantitative accumulation of CPEs along with preying on the green alga *Chlamydomonas*, where accumulation of cPPB-*b*E, chlorophyll-*b*-derived CPEs can be only explained by catabolic conversion of chlorophyll *b* produced in *Chlamydomonas* by the chrysophycean. Importantly, accumulation of CPEs was demonstrated in Cyano-TCs of the nonphototrophic chrysophyceans *Paraphysomonas* spp. and *Picophagus* sp. (Fig. 2e and Supplementary Fig. S2F), suggesting that CACC is indeed widely preserved at least among chrysophyceans. Such mixotrophy has also been known among those algae with secondary chloroplasts including chrysophyceans (ochrophytes), haptophytes, dinophytes, and cryptophytes [28]. Although it has not been thoroughly checked, the apparent absence of CACC in some of the UCs including those of cryptophytes may not reflect the lack of CACC at all, since we have not examined then under culture conditions as Cyano-TC. Obviously, therefore, investigation of CACC among algivorous mixotrophs is an important topic for future research.

That CACC among secondary phototrophs is derived from phagotrophic ancestors is supported by the consistent absence of CPEs among the green and red algae (Chloroplastida and Rhodophyceae, respectively; Fig. 1 and Supplementary Table S2). In fact, Archaeplastida does not exhibit accumulation of CPEs. However, it is unclear whether the phagotrophic ancestor of Archaeplastida (which acquired a cyanobacterial symbiont by phagocytosis) was a CPE producer, because phagocytosis is very unusual in extant Archaeplastida, with very few exceptions (mixotrophic green algae, such as *Cymbomonas* [29]). Nonetheless, Archaeplastida also requires a catabolic strategy to detoxify chlorophylls when attempting oxygenic photosynthesis, although this strategy might differ from the CACC. Among archaeplastids, land plants are known to catabolize chlorophylls into colorless and nonphototoxic catabolites [30, 31]. This metabolic process is called the phyllobilin/PaO pathway, after its key enzyme, pheophorbide *a* oxygenase (PaO), which oxidatively cleaves the robust chlorin structure of the chlorophyll derivative into various linear tetrapyrroles (phyllobilins). Sequences homologous to the *PAO* gene have been widely identified among the Chloroplastida and cyanobacteria, as well as other algae with secondary chloroplasts [32]. Although a similar function has not yet been identified for these homologs, the apparent lack of CACC in some of these organisms indicates that they may degrade chlorophyll to nonphototoxic colorless products (e.g., unconjugated phyllobilins). Associated with this study, for example, *Palpitomonas bilix*, the most basal lineage of Cryptista (Supplementary Fig. S2E), consumed a pedinophycean alga, but no CPE was detected (Supplementary Table S2). Furthermore, the green color of the chloroplasts of the pedinophycean rapidly faded to transparency under microscopic observation (Fig. 3f), suggesting the function of another as-yet-unknown type of chlorophyll catabolism in *P. bilix*. Therefore, the endosymbiosis of a cyanobacterium by the common ancestor of Archaeplastida, which gave rise to chloroplasts, might have been facilitated by a detoxification strategy other than the CPE accumulation.

The observed phylogenetic ubiquity of CACC among eukaryotes strongly indicates that the acquisition of CACC was a key evolutionary step that led to the diversity of extant eukaryotes. CACC is a “rate-emphasizing” process [6] that is rapid and requires no substantial biochemical cost and thus advantageous in digestive processing of algal materials. On the other hand, another “quality-emphasizing” process [6], such as the phyllobilin/PAO pathway, is rigorously but only slowly degrading chlorophylls with substantial biochemical costs (i.e., in the consumption of reducing cofactors and ATP); hence, it is less suited for algivorous processing. A typical example illustrating this can be found in the Rhizaria, in which accumulation of CPEs was detected in all 12 algivorous strains (representing 9 distinct lineages) and in all 12 phototrophic strains (chlorarachniophytes), encompassing the full rhizarian diversity. This strongly suggests that CACC is plesiomorphic in this MEA. Importantly, the evidence of CACC in Retaria demonstrates both its ecological and paleoecological significance through time. Foraminifers and radiolarians are major heterotrophs in modern oceans and represent up to 33% of large-zooplankton (>600 μm) communities [33]. Furthermore, these microeukaryotes form mineral skeletons or tests, which allows their preservation to be reliable fossil evidence. Many fossil occurrences of these organisms, which date back to the early Cambrian Period [34, 35] or earlier [36], support the consistent importance of the rhizarian heterotrophs, which constituted the primary consumers in the marine food web throughout the Phanerozoic. The strong conservation of CACC in this taxon suggests the broad importance of algivory in the evolution of the Rhizaria.

Why does CACC occur so widely among eukaryotes? Was the CACC found in different MEAs inherited through ancestor-descendent relationships? The wide occurrence of CACC reflects the fact that managing the phototoxicity of chlorophylls is crucial to any organism living in an illuminated, oxygenated environment and in close contact with chlorophyll-dependent photosynthesis. The consistent observation of CACC in particular clades (e.g., Rhizaria, Euglenozoa, and Dinoflagellata) suggests that it was inherited from the ancestors of these clades (Supplementary Fig. S2). Moreover, CACC frequently occurs among the basal lineages of most of MEAs, such as Stramenopiles, Haptista, and Cryptista (Supplementary Fig. S2). These observations strongly suggest that the evolutionary origin(s) of CACC can be traced back to the early eukaryote radiation, if not to LECA. Another hypothesis is that CACC have been acquired several times in the early stage of eukaryote radiation; in such a case, CACC may have been spread horizontally by horizontal gene transfer, or acquired independently several times through the course of evolution. Unfortunately, however, precise reconstruction of the evolutionary history of CACC is currently difficult because the genetic basis for CACC still remains unknown.

**Fig. 4:**
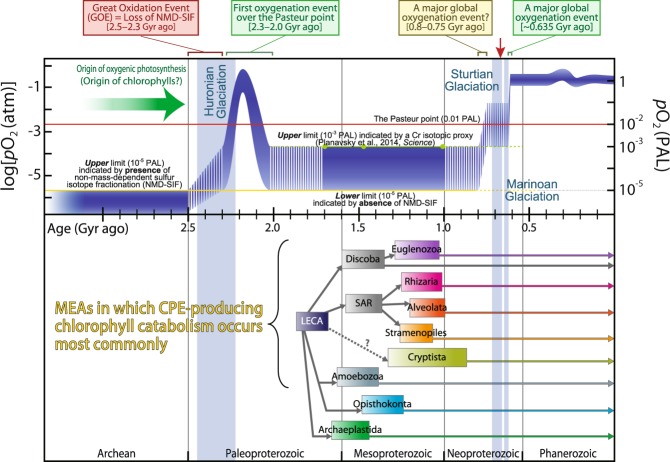
Comparative reconstruction of the temporal evolution of atmospheric *p*O_2_ and estimated age of emergences of major eukaryotic assemblages (MEAs). In the upper diagram, the blue band shows the estimated range of Earth’s atmospheric oxygen content for the last three billion years (Gyr), modified from Lyons et al. [2]. The yellow and green lines delineate the upper and lower limits, respectively, of the estimated range based on geochemical proxies [3]. Red arrow on the top indicates the time point (0.80–0.64 Gyr ago) when *p*O_2_ exceeded the Pasteur point (the presumed level of oxygen required for mitochondrial respiration). In the lower diagram, the estimated divergence times (95% highest probability density) of selected MEAs and the estimated age of LECA are shown, according to Parfrey et al. [37]. This illustrates a conspicuous discrepancy between the timing of the final oxygenation of the atmosphere and the appearance of extant eukaryotic lineages with notable affinity for molecular oxygen. PAL present atmospheric level, MEAs major eukaryotic assemblages, LECA last eukaryotic common ancestor, SAR the supergroup Stramenopiles–Alveolata–Rhizaria

No matter what process spread CACC across branches of extant eukaryotes, it is very likely that CACC has already been established by the earliest Neoproterozoic (Fig. 4). Convincing examples are found in Rhizaria and Euglenozoa, and imply the universal occurrence of CACC, because the origins of these groups have been dated to the late Mesoproterozoic (ca. 1017–1256 Ma and ca. 1030–1290 Ma, respectively), according to temporal reconstructions based on molecular clock analyses [37–39]. Therefore, CACC is estimated to have been acquired by eukaryotes before the last Snowball Earth event, and before the increase in global *p*O_2_.

The evolution of algivores equipped with CACC must have been an ecological breakthrough in the history of the eukaryotes. Regardless of the ambient atmospheric *p*O_2_ at that time, CACC must have evolved to allow microeukaryotes to prey on ancestral phototrophs that generated oxygen (cyanobacteria and/or eukaryotic algae with primary chloroplasts). This explanation is consistent with the origin of mitochondrial respiration, which can also be traced back at least to LECA (Fig. 4). CACC subsequently allowed the direct and massive in situ consumption (i.e., in the presence of light [6]) of the overwhelmingly important oxygenic primary producers in the water column that were responsible for late Proterozoic global oxygenation (Fig. 4). Thus, the ecological advantage conferred by CACC exapted extant eukaryotic lineages to the dramatic biogeochemical changes in primary production that led to the increase in global *p*O_2_, and together with the physiological advantages conferred by mitochondria, allowed their construction of, and successful radiation into, the fully oxygenated Earth to the present day.

## Supplementary information

Supplementary Table S1

Supplementary Table S2

Supplementary Information for: Taming chlorophylls by early eukaryotes underpinned algal interactions and the diversification of the eukaryotes on the oxygenated Earth
