# Supplementary material for: Taming chlorophylls by early eukaryotes underpinned algal interactions and the diversification of the eukaryotes on the oxygenated Earth
Source: ISME J. 2019 Feb 26;13(8):1899–910. doi: 10.1038/s41396-019-0377-0 (PMC6775998; doi:10.1038/s41396-019-0377-0)
Supplement: Supplementary file 3 — Supplementary Information for: Taming chlorophylls by early eukaryotes underpinned algal interactions and the diversification of the eukaryotes on the oxygenated Earth [file 41396_2019_377_MOESM3_ESM.pdf]

Supplementary Information for:

Taming chlorophylls by early eukaryotes underpinned algal interactions and the diversification of the eukaryotes on the oxygenated Earth

Yuichiro Kashiyama[1,2,3,*], Akiko Yokoyama[4,5], Takashi Shiratori[4,6], Sebastian Hess[7], Fabrice Not[8], Charles Bachy[8], Andres Gutierrez-Rodriguez[8], Jun Kawahara[1], Toshinobu Suzaki[9], Masami Nakazawa[10], Takahiro Ishikawa[11], Moe Maruyama[1], Mengyun Wang[12], Man Chen[12], Yingchun Gong[12], Kensuke Seto[13,14], Maiko Kagami[13,14], Yoko Hamamoto[15,16], Daiske Honda[16,17], Takahiro Umetani[2], Akira Shihongi[1], Motoki Kayama[1], Toshiki Matsuda[1], Junya Taira[2], Akinori Yabuki[6], Masashi Tsuchiya[6], Yoshihisa Hirakawa[4], Akane Kawaguchi[18], Mami Nomura[18,19], Atsushi Nakamura[18], Noriaki Namba[18], Mitsufumi Matsumoto[20], Tsuyoshi Tanaka[21], Tomoko Yoshino[21], Rina Higuchi[9], Akihiro Yamamoto[2], Tadanobu Maruyama[1], Aika Yamaguchi[22], Akihiro Uzuka[23], Shinya Miyagishima[23], Goro Tanifuji[24], Masanobu Kawachi[25], Yusuke Kinoshita[3], and Hitoshi Tamiaki[3]

[1]Graduate School of Engineering, Fukui University of Technology, Fukui, Fukui 910-8505, Japan.

[2]Department of Environmental and Biological Chemistry, Faculty of Engineering, Fukui University of Technology, Fukui, Fukui 910-8505, Japan.

[3]Graduate School of Life Sciences, Ritsumeikan University, Kusatsu, Shiga 525-8577, Japan.

[4]Faculty of Life and Environmental Sciences, University of Tsukuba, Tsukuba, Ibaraki 305-8572, Japan.

[5]Center for Regional Environmental Research, National Institute for Environmental Studies, Tsukuba, Ibaraki 305-8506, Japan.

[6]Department of Marine Biodiversity Research, Japan Agency for Marine–Earth Science and Technology, Yokosuka, Kanagawa 237-0061, Japan.

[7]Life Sciences Centre, Dalhousie University, Halifax, Nova Scotia B3H 4R2, Canada.

[8]Sorbonne University, CNRS, UMR7144, Ecology of Marine Plankton Team, Station Biologique de Roscoff, 29680 Roscoff, France.

[9]Institute of Hydrobiology, Chinese Academy of Sciences, Wuchang District, Wuhan 430072, China.

[10]Department of Environmental Science, Faculty of Science, Toho University, Funabashi, Chiba 274-8510, Japan.

[11]Graduate School of Environment and Information Sciences, Yokohama National University, Yokohama, Kanagawa 240-8502, Japan.

[12]Graduate School of Natural Science, Konan University, Kobe, Hyogo 658-8501, Japan.

[13]Institute for Integrative Neurobiology, Konan University, Kobe, Hyogo 658-8501, Japan.

[14]Faculty of Science and Engineering, Konan University, Kobe, Hyogo 658-8501, Japan.

[15]Graduate School of Life and Environmental Sciences, University of Tsukuba, Tsukuba, Ibaraki 305-8572, Japan.

[16]Graduate School of Science, Kyoto University, Kyoto 606-8502, Japan.

[17]Biotechnology Laboratory, Electric Power Development Co., Ltd, Kitakyusyu, Fukuoka 808-0111, Japan.

[18]Institute of Engineering, Tokyo University of Agriculture and Technology, Koganei, Tokyo 184-8588, Japan.

[19]Graduate School of Science, Kobe University, Kobe, Hyogo 657-8501, Japan.

[20]Kobe University Research Center for Inland Seas, Kobe, Hyogo 657-8501, Japan.

[21]Department of Cell Genetics, National Institute of Genetics, Mishima, Shizuoka 411-8540, Japan.

[22]Division of Applied Life Sciences, Graduate School of Life and Environmental Sciences, Osaka Prefecture University, Sakai, Osaka 599-8531, Japan.

[23]Faculty of Life and Environmental Science, Department of Life Science and Biotechnology, Shimane University, Matsue, Shimane 690-8504, Japan.

[24]National Museum of Nature and Science, Tsukuba, Ibaraki 305-0005, Japan.

[25]Center for Environmental Biology and Ecosystem Studies, National Institute for Environmental Studies, Tsukuba, Ibaraki 305-8506, Japan.

[*]Corresponding author. Email: chiro@fukui-ut.ac.jp

This PDF file includes:

Supplementary Fig. S1–S5
Supplementary Table S1 to S2
References (1–28)

References cited in Supplementary Figs and Tables

1. Koren LE, Hutner SH. High yielding media for photosynthesising *Euglena gracilis* z. J Protozool. 1967;14 Suppl.:17.
2. Cramer M, Myers J. Growth and photosynthetic characteristics of *Euglena gracilis*. Arch Mikrobiol. 1952;17:384.
3. Goericke R, Strom SL, Bell MA. Distribution and sources of cyclic pheophorbides in the marine environment. Limnol Oceanogr. 2000;45:200–211.
4. Yamada N, Tanaka A, Horiguchi T. cPPB-aE is discovered from photosynthetic benthic dinoflagellates. J Phycol. 2014;50:101–107.
5. Suzuki T, Casareto BE, Shioi Y. Finding of 132,173-cyclopheophorbide *a* enol as a degradation product of chlorophyll in shrunk zooxanthellae of the coral Montipora digitata. J Phycol. 2015;51:37–45.
6. Kashiyama Y, Yokoyama A, Kinoshita Y, Shoji S, Miyashiya H, Shiratori T, et al. Ubiquity and quantitative significance of detoxification catabolism of chlorophyll associated with protistan herbivory. Proc Natl Acad Sci USA. 2012;109:17328–17335.
7. Kinoshita Y, Kayama M, Kashiyama Y, Tamiaki H. *In vivo* and *in vitro* preparation of divinyl-13^2,17^3-cyclopheophorbide-*a* enol. Bioorg Med Chem Lett. 2018;28:1090–1092.
8. Adl SM, Bass D, Lane CE, Lukeš J, Schoch CL, Smirnov A, et al. Revisions to the classification, nomenclature, and diversity of eukaryotes. J Eukaryot Microbiol. 2018;66:4–119.
9. Fukuda SY, Iwamoto K, Atsumi M, Yokoyama A, Nakayama T, Ishida K, et al. Global searches for microalgae and aquatic plants that can eliminate radioactive cesium, iodine and strontium from the radio-polluted aquatic environment: a bioremediation strategy. J Plant Res. 2014;127:79–89.
10. Yamaguchi A, Yubuki N, Leander BS. Morphostasis in a novel eukaryote illuminates the evolutionary transition from phagotrophy to phototrophy: description of *Rapaza viridis* n. gen. et sp. (Euglenozoa, Euglenida). BMC Evol Biol. 2012;12:29.
11. Yabuki A, Tame A. Phylogeny and reclassification of *Hemistasia phaeocysticola* (Scherffel) Elbrächter & Schnepf, 1996. J Eukaryot Microbiol. 2015;62:426–429.
12. Hirakawa Y, Howe A, James ER, Keeling PJ. Morphological diversity between culture strains of a chlorarachniophyte, *Lotharella globosa*. PLoS ONE. 2011;6:e23193.
13. Shiratori T, Yokoyama A, Ishida K. Phylogeny, ultrastructure, and flagellar apparatus of a new Marimonad flagellate *Abollifer globosa* sp. nov. (Imbricatea, Cercozoa). Protist. 2014;160:808–824.
14. Hess S, Melkonian M. The mystery of clade X: *Orciraptor* gen. nov. and *Viridiraptor* gen. nov. are highly specialised, algivorous amoeboflagellates (Glissomonadida, Cercozoa). Protist. 2013;164:706–747.
15. Gong Y, Patterson DJ, Li Y, Hu Z, Sommerfeld M, Chen Y, et al. *Vernalophrys algivore* gen. nov., sp. nov. (Rhizaria: Cercozoa: Vampyrellida), a new algal predator isolated from outdoor mass culture of *Scenedesmus dimorphus*. Appl Environ Microbiol. 2015;81:3900–3913.
16. Yokoyama A, Shiratori T, Inouye I, Kinoshita Y, Mizoguchi T, et al. 13^2,17^3-Cyclopheophorbide *b* enol as a catabolite of chlorophyll *b* in phycophagy by protists. FEBS Lett. 2013;587:2578–2583.
17. Yabuki A, Kamikawa R, Ishikawa SA, Kolisko M, Kim E, Tanabe AS et al. *Palpitomonas bilix* represents a basal cryptist lineage: insight into the character evolution in Cryptista. Sci Rep. 2014;4:4641.
18. Dieckhoff HS, Freiburg M, Heckmann K. The isolation of gamones 3 and 4 of *Euplotes octocarinatus*. Eur J Biochem. 1987;168:89–94.
19. Wang QY, Zhang Y, Li AF, Zhang CW. Effects of sulfur concentration on the photosynthetic physiology and biochemical composition of *Scenedesmus acuminatus*. Acta Hydrobiol Sinica. 2017;41:904–913.
20. Ma M, Yuan D, He Y, Park M, Gong Y, Hu Q. Effective control of *Poterioochromonas malhamensis* in pilot-scale culture of *Chlorella sorokiniana* GT-1 by maintaining CO_2-mediated low culture pH. Algal Res. 2017;26:436–444.
21. Takaichi S, Yokoyama A, Mochimaru M, Uchida H, Murakami A. Carotenogenesis diversification in phylogenetic lineages of Rhodophyta. J Phycol. 2016;52:329–338.
22. Yokoyama A, Hara Y. Distribution and composition of UV-absorbing compounds, mycosporine-like amino acids in the Porphyridiales (Rhodophyta), with special reference to their taxonomic and phylogenetic significance. Bul Yamagata Univ Nat Sci. 2008;16:113–125.
23. Sekimoto S., Klochkova TA, West JA, Beakes GW, Honda D. *Olpidiopsis bostrychiae* sp. nov.: an endoparasitic oomycete that infects *Bostrychia* and other red algae (Rhodophyta). Phycologia. 2009;48:460–472.

24. Matsumoto M, Mayama S, Nemoto M, Fukuda Y, Muto M, Yoshino T, et al. Morphological and molecular phylogenetic analysis of the high triglyceride-producing marine diatom, *Fistulifera solaris* sp. nov. (Bacillariophyceae). Phycol Res. 2014;62:257–268.

25. Shiratori T, Thakur R, Ishida K. *Pseudophyllomitus vesiculosus* (Larsen and Patterson 1990) Lee, 2002, a poorly studied phagotrophic biflagellate is the first characterized member of stramenopile environmental clade MAST-6. Protist. 2017;168:439–451.

26. Honda D, Yokochi T, Nakahara T, Raghukumar S, Nakagiri A, Schaumann K, et al. Molecular phylogeny of labyrinthulids and thraustochytrids based on the sequencing of 18S ribosomal RNA gene. J Eukaryot Microbiol. 1999;46:637–647.

27. Shiratori T, Nakayama T, Ishida K. A new deep-branching stramenopile, *Platysulcus tardus* gen. nov., sp. nov. Protist. 2015;166:337–348.

28. Yabuki A, Eikrem W, Takishita K, Patterson DJ. Fine structure of *Telonema subtilis* Griessmann, 1913: a flagellate with a unique cytoskeletal structure among eukaryotes. Protist. 2013;164:556–569.

29. Seto K, Kagami M, Degawa Y. Phylogenetic position of parasitic chytrids on diatoms: characterization of a novel clade in Chytridiomycota. J Eukaryot Microbiol. 2017;64:383–393.

Supplementary Figs and Tables

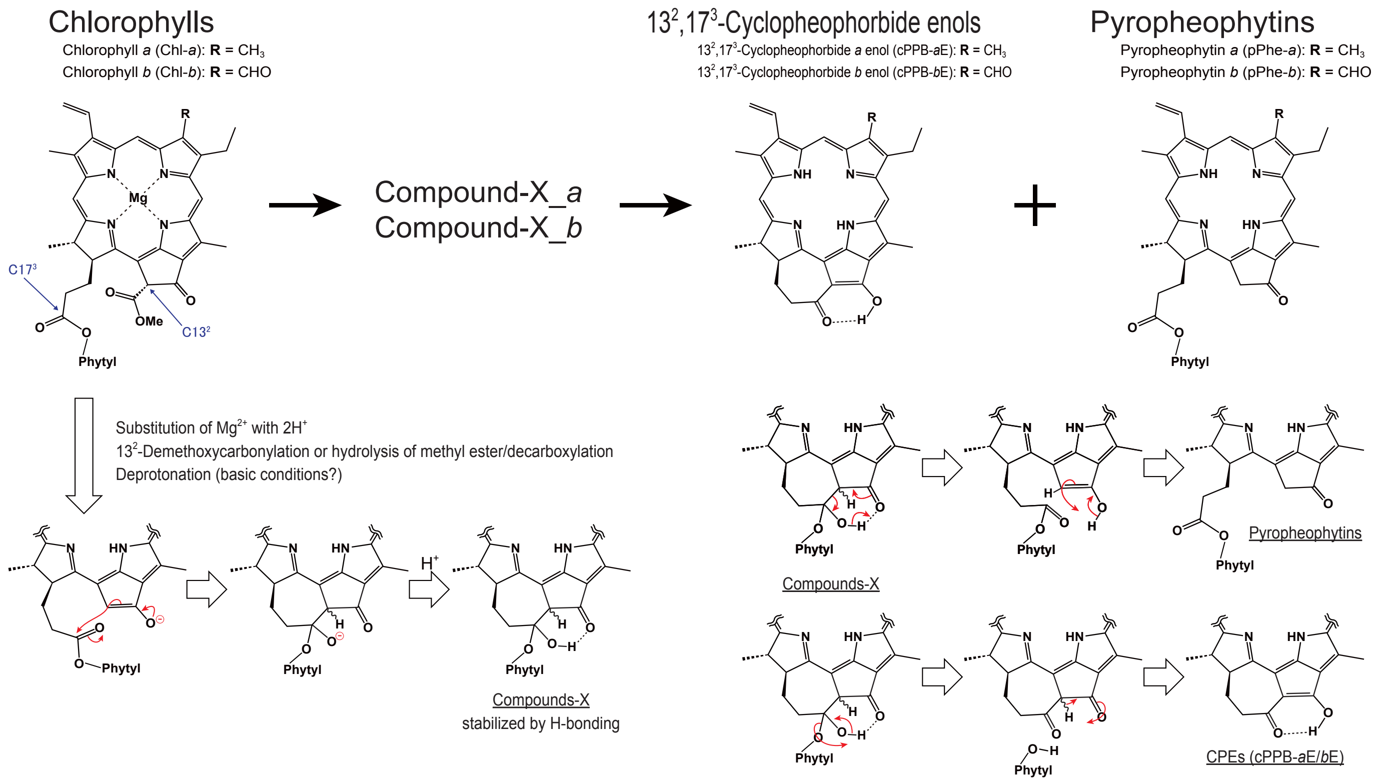

Supplementary Fig. S1 Chemical structures of chlorophyll derivatives discussed in the present work, with which was constituted a schematic diagram of a possible metabolic path of CPE-accumulating chlorophyll catabolism. When CPEs are present, compounds-X and pyropheophytins were also detected in certain ratios that were highly variable depending on the strain examined. In most of case, compounds-X and pyropheophytins were very minor components but often very abundant particularly among phototrophic euglenids (euglenophytes), such as a freshwater euglenophyte *Euglena gracilis*. A high-resolution mass spectrum of compound-X_*a* was found m/z = 813.5701 by after chromatographic isolation using the analytical HPLC with a fraction collector (Fig. 2). When the isolated fraction of compound-X_*a* was re-injected to the identical analytical HPLC, additional peaks of cPPB-*a*E and pyropheophytin *a* were observed at very different retention times from that of compound-X_*a*, suggesting that compounds-X were very unstable intermediates that could generate both CPEs and pyropheophytins. Altogether, the structures of compounds-X and hence a metabolic path are deduced as illustrated.

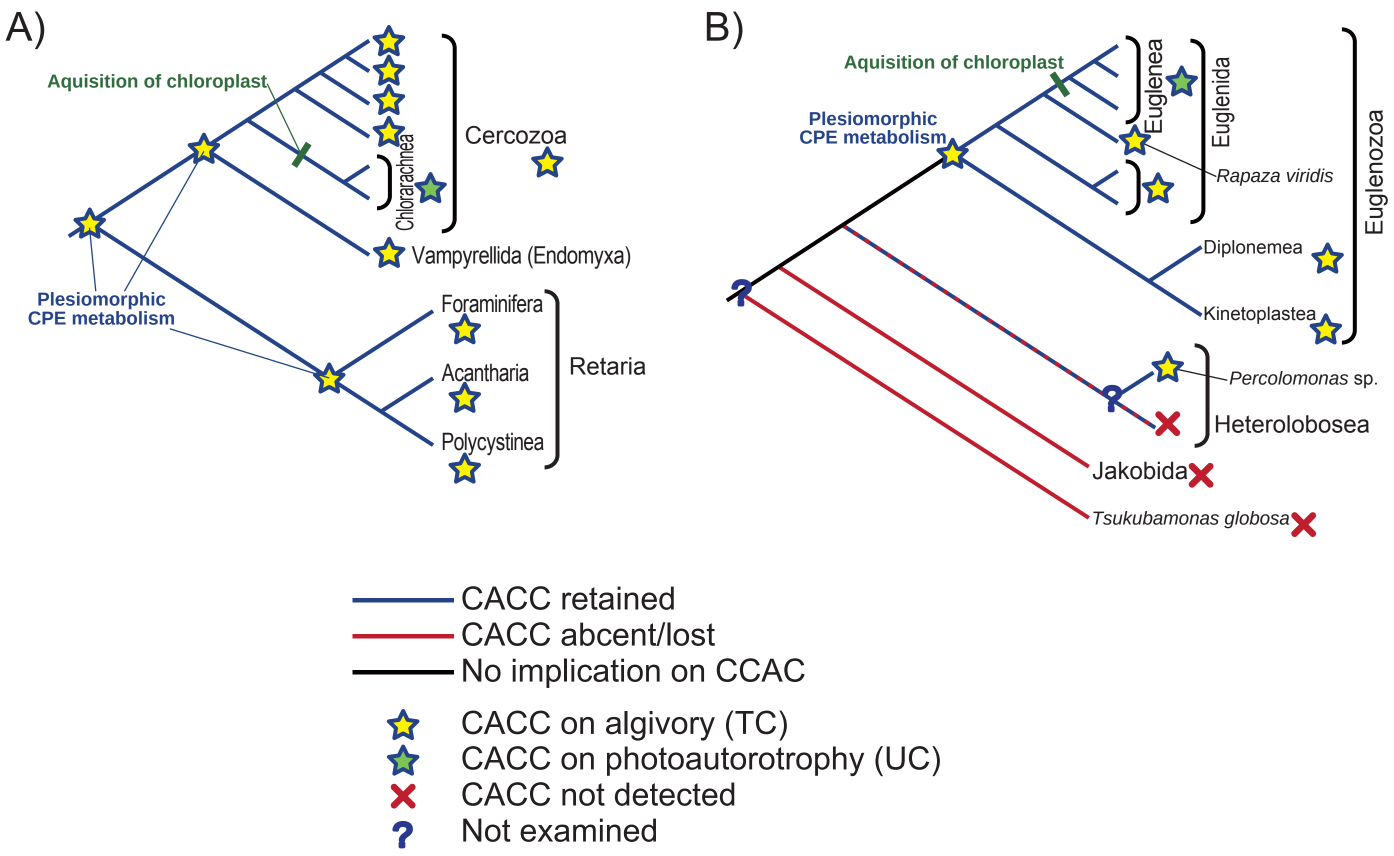

Supplementary Fig. S2 Schematic cladograms of each MEA that involves microeukaryotes conducting CPE-accumulating chlorophyll catabolism (CACC), illustrating possible evolutionary contexts of CACC. **a** Rhizaria, demonstrating accumulation of CPEs by both algivores and photoautotrophs without exception. **b** Discoba, in which CACC is general among one of the major clade Euglenozoa containing both algivores and phototrophs. **c** Alveolata, in which CACC is common among one of the major clade Dinophyceae containing both algivores and phototrophs (but with some exceptions among the phototrophs) but is rather sporadic among a deep-branched clade Ciliophora. **d** Haptista, comprising a derivative phototrophic group Haptophyta that generally does not accumulate CPEs (but a few exceptions) and basally-branched algivorous group Centroplasthelida (centroheliozoans) that accumulates CPEs after ingestion of algal preys. **e** Cryptista, in which CACC is general among algivores but absent in phototrophs, consisting of the most derivative clade, as well as the most basally-branched species *Palpitomonas bilix*. **f** Stramenopiles, in which more derivative groups including phototrophic Ochrophyta generally does not accumulate CPE but more basal groups tend to exhibit accumulation of CPEs upon algivory.

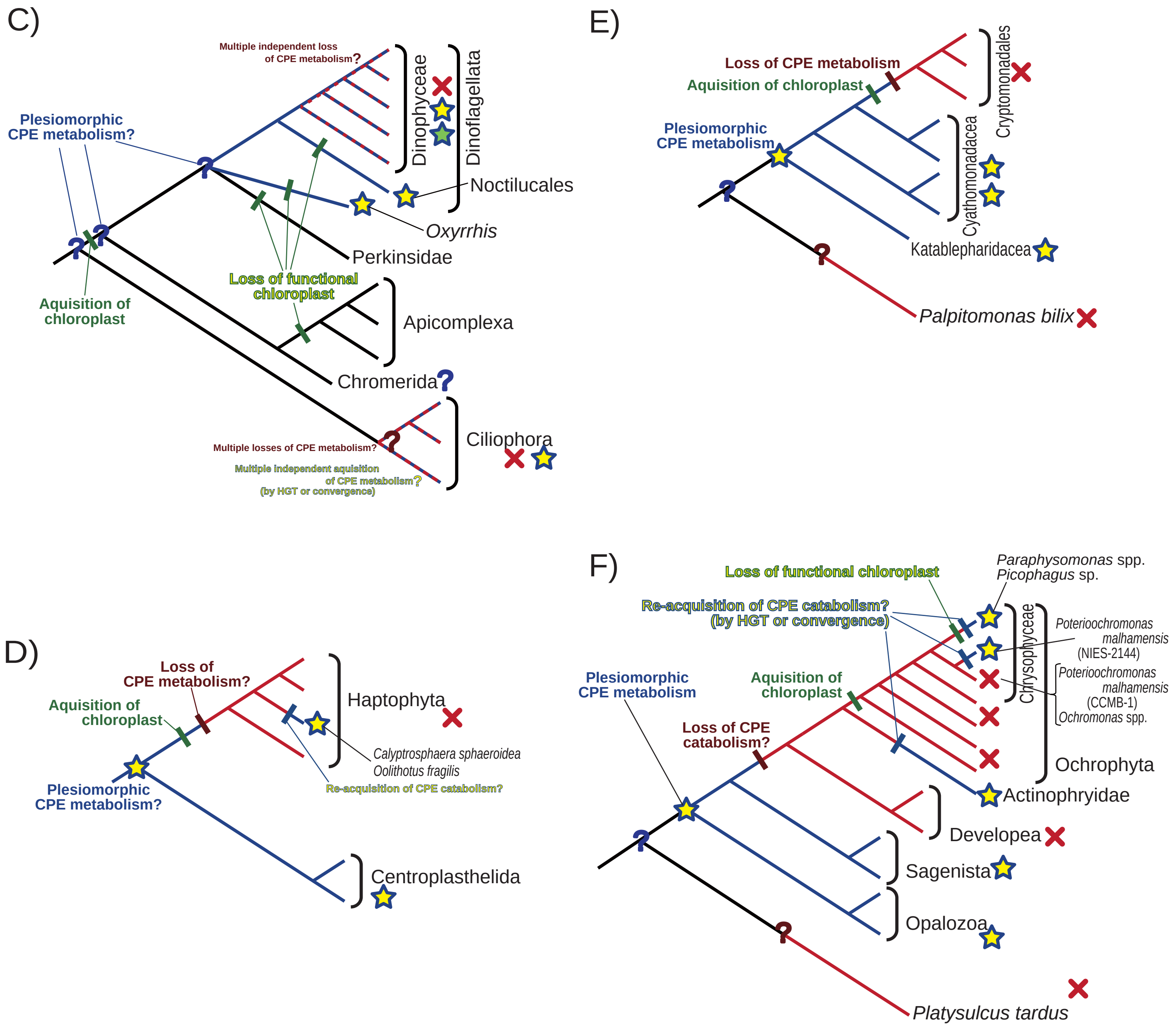

Supplementary Fig. S2 (continued)

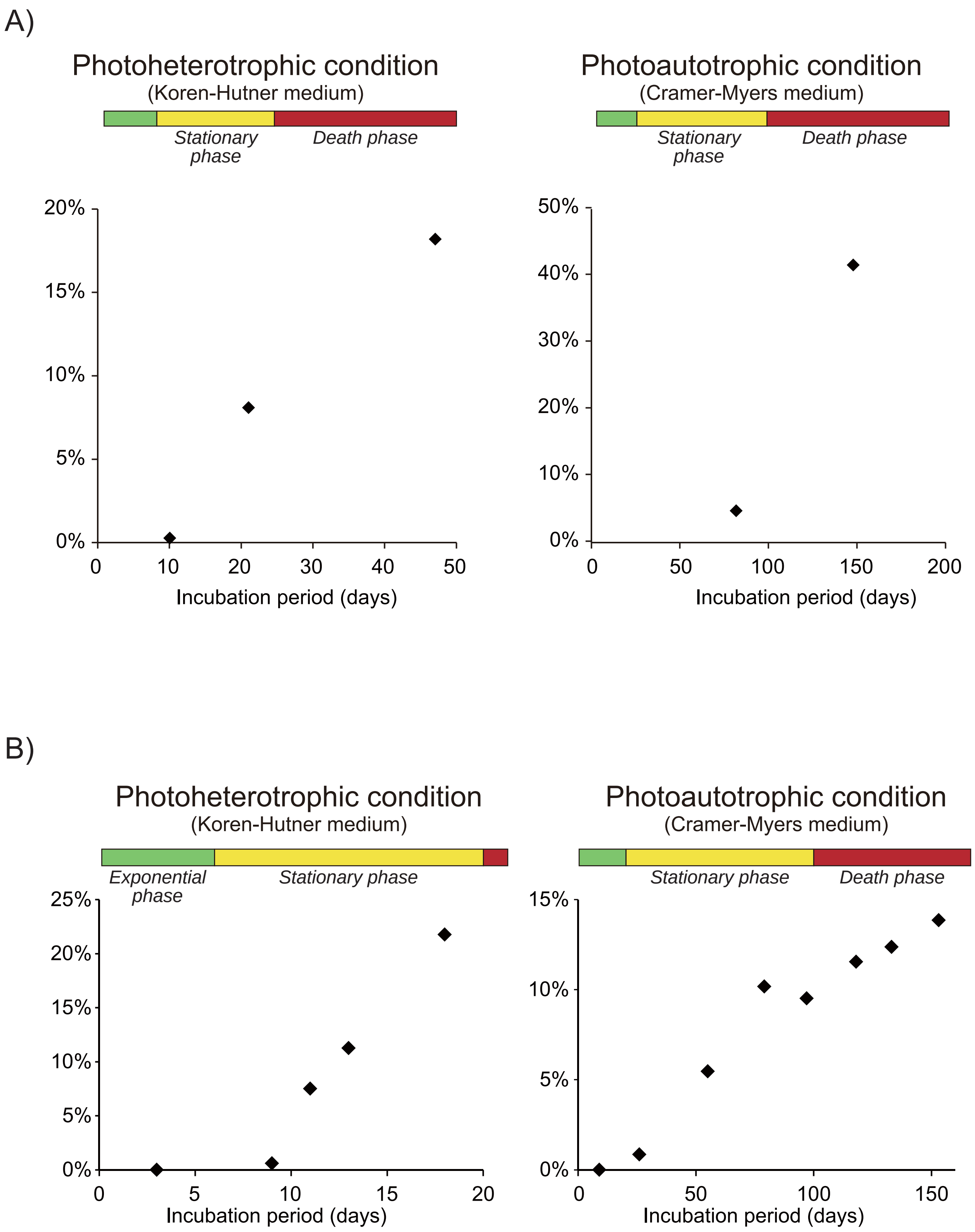

Supplementary Fig. S3 Accumulations of CPEs in aged cultures of *Euglena gracilis*. cPPB-*a*E (a CPE derived from Chl-*a*) and its related compounds (metabolic intermediate compound-X_*a* and a byproduct pPhe-*a*; see Supplementary Fig. S1) was insignificant in cultures of *E. gracilis* (Strain Z) during exponential growth phases in both photomixotrophic conditions with medium containing organic substrates for osmotrophy (i.e., Koren-Hutner medium) [1] and photoautotrophic cultures (i.e., Cramer-Myers medium) [2]. A) Experiments conducted during 2013 with continuous light at 27°C; B) experiments conducted during 2018 with the light-dark cycle at 27°C.

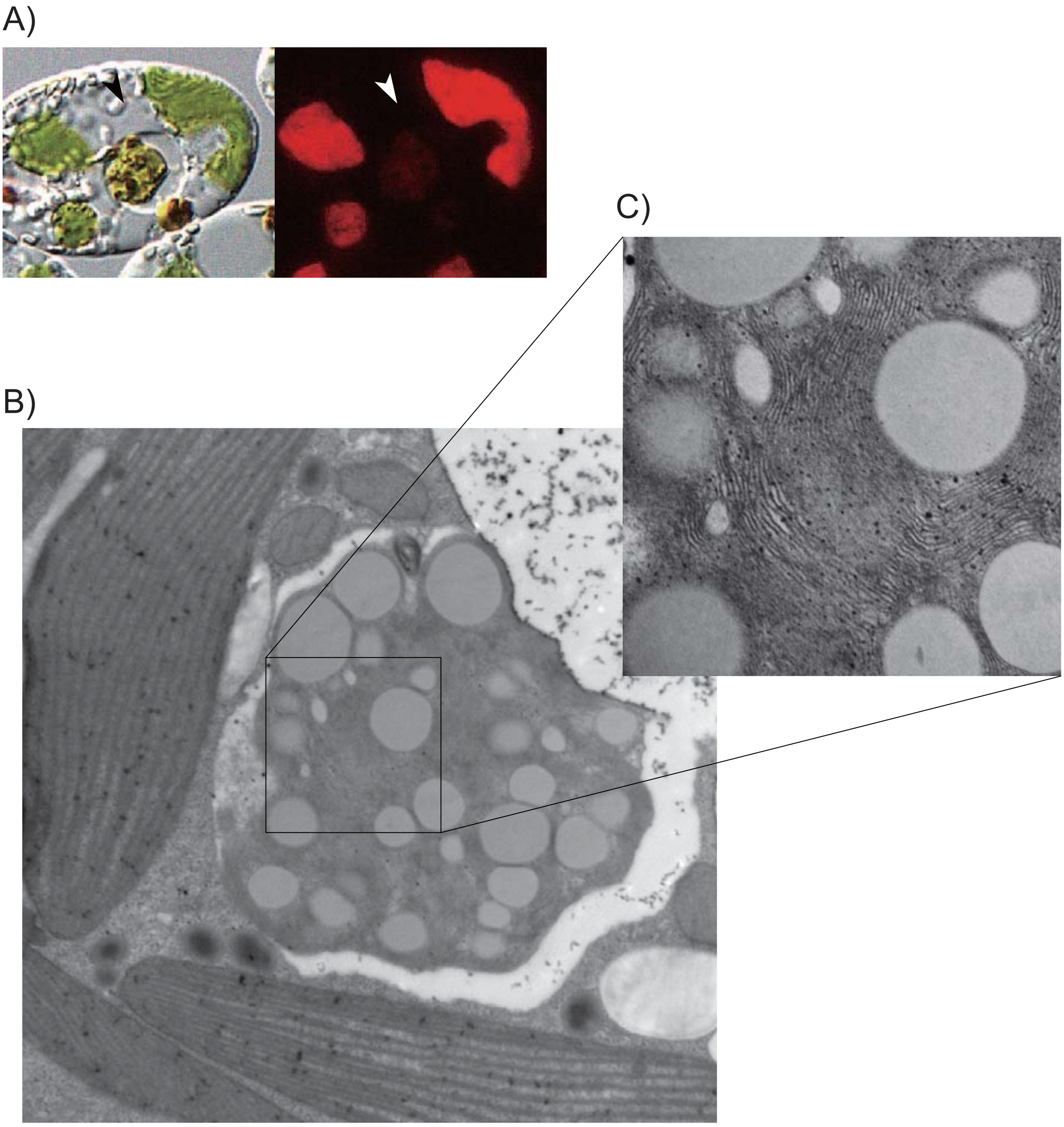

Supplementary Fig. S4 Microscopic observations of *Euglena gracilis* in an aged culture. A) A differential interference (left) and fluorescent images (excitation light, 400–440 nm) (right) of *E. gracilis*, exhibiting apparent dismantling of a chloroplast leading to formation of a brown globule. Note that chlorophyll autofluorescence had been nearly lost in a pale chloroplast in relatively early stage of the chlorophyll dismantling (arrowhead), indicating catabolic conversion of chlorophylls into CPEs. B) A transmission electron microscopic image and C) its extended view of an intracellular structure corresponding to the dismantled chloroplast, exhibiting formation of lipid bodies surrounded by bundle of membranes that are probable remnant structures of degraded thylakoids.

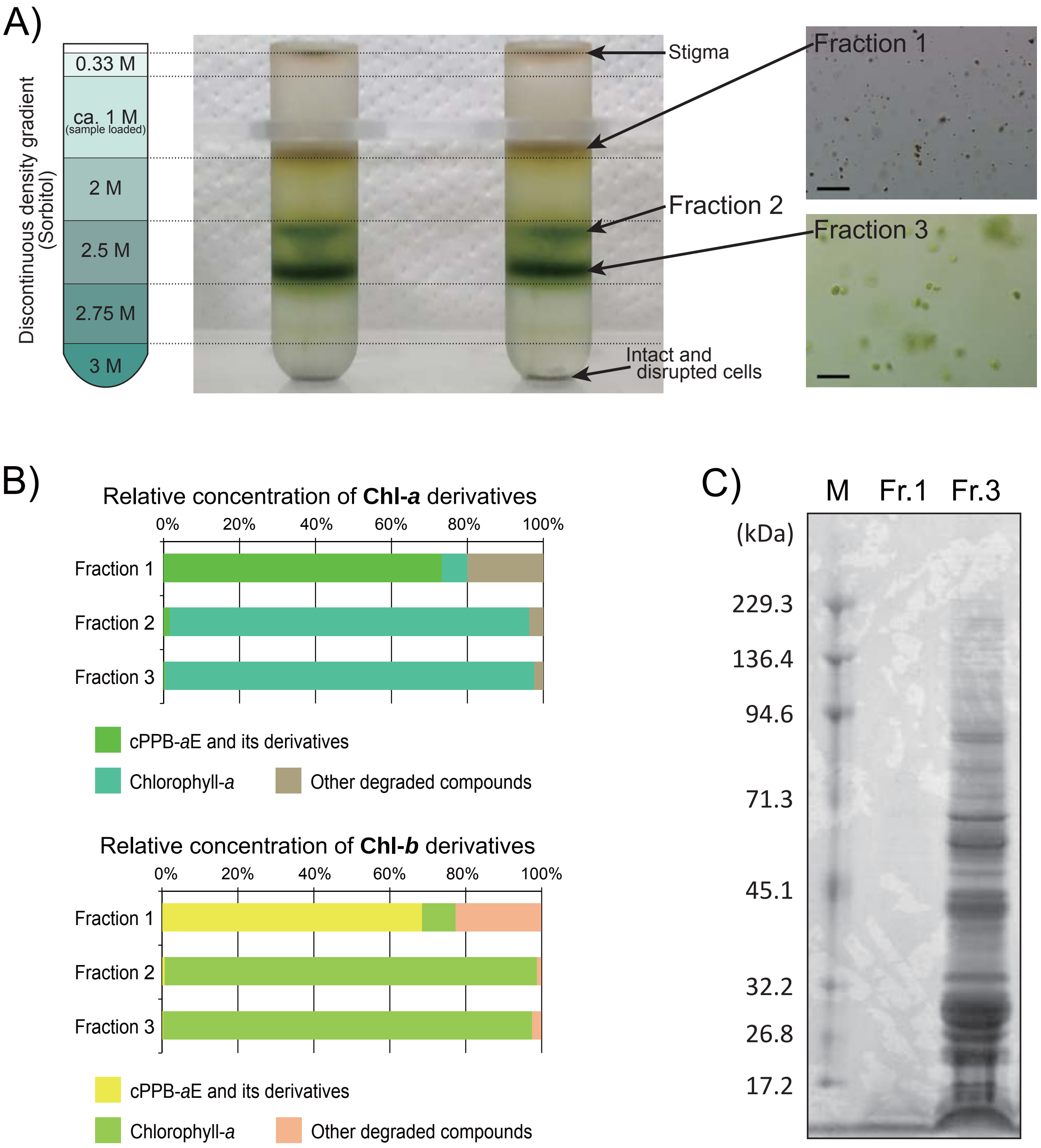

Supplementary Fig. S5 Isolation of green intact chloroplasts and brown globules from cells of *Euglena gracilis* strain-z in a photoautotrophic culture (Cramer-Myers medium) in a late stationary phase of growth and their characterizations. A) Green intact chloroplasts and brown globules were isolated by a discontinuous sorbitol density gradient ultracentrifugation. Fraction 1 exclusively contained the brown globules, whereas fractions 2 and 3 contained disrupted and intact chloroplasts; the quality of each fraction was confirmed by high magnification DIC microscopy (scale bars: 20 µm). B) Relative concentrations of chlorophyll derivatives in each isolated fraction. Fraction 1 was highly enriched in CPEs and its related compounds (Supplementary Fig. S1) and depleted in chlorophylls, whereas fraction 2 and 3 contained intact chlorophylls exclusively. C) Proteins were nearly absent in the brown globule of *E. gracilis* formed from chloroplasts. SDS-PAGE of *E. gracilis* proteins extracted from the grown globules (Fr. 1) and from intact chloroplasts (Fr.3) (M: size marker). The proteins in the gel were stained with Coomassie Brilliant Blue. It indicates that proteins of chloroplasts were degraded for recycling; therefore, the process of formation of brown globules from chloroplasts in *E. gracilis* is indeed ordered dismantling of the organelle, which must be accompanied with a strategic degradation of the chlorophylls contained therein.

Supplementary Table S1 An HPLC gradient programs for ternary solvent system used in the present study.

Time (min)	Solvent			Note
	A (%)	B (%)	C (%)	
0.0 → 10.0	100 → 0	0 → 100	0	Actual analysis
10.0 → 25.0	0	100 → 0	0 → 100	
25.0 → 25.5	0	0 → 100	100 → 0	Re-equilibration
25.5 → 26.0	0 → 100	100 → 0	0	
26.0 → 35.0	100	0	0	

Solvents: A, acetonitrile: a 0.2 M aqueous solution of imidazole with trifluoroacetic acid (TFA) (pH = 5.2) = 1:1; B, acetonitrile: ethyl acetate: a 1 M aqueous solution of imidazole with TFA (pH = 5.2) = 80:10:10; C, a 7:3 mixture of acetonitrile and ethyl acetate solution with 0.1 M imidazole.

Supplementary Table S2 List of strains examined in the present study with results on detection of CPEs in their cultural experiments, as well as species reported in the previous studies on productions of CPEs (*Goericke et al. [3]; **Yamada et al. [4]; ***Suzuki et al. [5]; §Kashiyama et al. [6]; §§Kinoshita et al. [7]). In the column of Culture type, UC, TC, and Cyano-TCs refer to an unialgal culture, a two-membered culture, and a cyano-bacterivorous two-membered culture, respectively. The classification and nomenclatures of eukaryotic taxa are based on Adl et al. [8].

Taxonomy					Species	Strain ID (isolator)	Culture type	Axenic	Prey algal strains (strain ID and/or isolator)	CPEs	Note
Eukaryota	Discoba	Euglenozoa	Euglenida	Euglenea	*Colacium* sp.	AKI001 (A. Yamamoto)	UC	†	—	Detected	Unpublished strain
					Euglena gracilis	Strain-Z	UC	†	—	Detected	
					Euglena mutabilis	NIES-286	UC	†	—	Detected	
					Euglena viridis	NIES-2149	UC	†	—	Detected	
					Euglena sp.	INB092 (A. Yokoyama)	UC		—	Detected	Fukuda et al. [9]
					Euglena sp.	INB114 (A. Yokoyama)	UC		—	Detected	Unpublished strain
					Euglenaria anabaena	INB108 (A. Yokoyama)	UC		—	Detected	Fukuda et al. [9]
					Euglenaria caudata	INB033 (A. Yokoyama)	UC		—	Detected	Unpublished strain
					Phacus pleuronectes	INB077 (A. Yokoyama)	UC		—	Detected	Unpublished strain
					Eutreptia sp.	CCAC 1914 B	UC		—	Detected	
					Eutreptiella eupharyngea	DB14 (A.Kawaguchi)	UC		—	Detected	Unpublished strain
					Eutreptiella eupharyngea	OS18 (A. Kawaguchi)	UC		—	Detected	Unpublished strain
					Eutreptiella gymnastica	CCMP1594	UC		—	Detected	
					Eutreptiella gymnastica	DB06 (A. Kawaguchi)	UC		—	Detected	Unpublished strain
					Eutreptiella gymnastica	NIES-381	UC		—	Detected	
					Eutreptiella gymnastica	SCCAP K-0334	UC		—	Detected	
					Eutreptiella sp.	CCMP389	UC		—	Detected	
					Eutreptiella sp.	NIES-2305	UC		—	Detected	
					Eutreptiella sp.	NIES-2325	UC		—	Detected	
					Rapaza viridis	(A. Yamaguchi)	TC		*Tetraselmis* sp. (A. Yamaguchi)	Detected	Yamaguchi et al. [10]
				Heteronematina	*Peranema* sp.	SRT225 (T. Shiratori)	TC		*Nitzschia sp.* (E06; T. Shiratori)	Detected	Unpublished strain
					Peranama trichophorum	FAL010 (T. Maruyama)	TC		*Synedra* sp. (TAU-kk; T. Umetani)	Detected	Unpublished strain
			Kinetoplastea	Metakinetoplastina	*Bodo saltans*	NIES-1439	Cyano-TC		*Synechococcus leopoliensis* (NIES-3277)	Detected	New prey-predator relationship
			Diplonemea	Diplonemida	*Hemistasia phaeocysticola*	NIES-3356	TC		*Thalassiosila* sp. (A. Yabuki)	Detected	Yabuki & Tame [11]
		Heterolobosea	Vahlkampfiidae		*Naegleria* sp.	(A. Uzuka)	Cyano-TC		*Synechococcus elongatus* (PCC 7942)	N.D.	Unpublished strain
					Paravahlkampfia sp.	MZD001 (T. Matsuda)	TC		*Synechococcus elongatus* (PCC 7942)	N.D.	Unpublished strain
					Euplaesiobystra sp.	EVE001 (J. Taira, *et al*.)	TC		*Fistulifera solaris* (T. Tanaka)	N.D.	Unpublished strain
					Percolomonas sp.	NIES-1441	Cyano-TC		*Prochlorococcus* sp. (NIES-2882)	Detected	New prey-predator relationship
		Jakobida	Histionina		*Reclinomonas americana*	NN101 (N. Namba)	Cyano-TC		*Prochlorococcus marinus* (NIES-2087)	N.D.	Unpublished strain
		Tsukubamonadida			*Tsukubamonas globosa*	NIES-1390	Cyano-TC		*Synechococcus leopoliensis* (NIES-3277)	N.D.	New prey-predator relationship
	Rhizaria	Cercozoa	Chlorarachnea (chlorarachniophytes)		*Amorphochlora amoebiformis*	CCMP2058	UC		—	Detected	
					Bigelowiella natans	NIES-2677	UC		—	Detected	
					Chlorarachnion reptans	CCCM449	UC		—	Detected	
					Chlorarachnion reptans	NIES-624	UC		—	Detected	
					Chlorarachnion reptans	NIES-1408	UC		—	Detected	
					Gymnochlora sp.	CCMP2014	UC		—	Detected	
					Lotharella globosa var. globosa	CCCM811	UC		—	Detected	
					Lotharella globosa var. fortis	Lex01 (Y. Hirakawa)	UC		—	Detected	Hirakawa et al. [12]
					Lotharella oceanica	CCMP622	UC		—	Detected	
					Lotharella reticulosa	NIES-2584	UC		—	Detected	
					Norrisiella sphaerica	NIES-2433	UC		—	Detected	
					(unidentified)	NIES-2502	UC		—	Detected	
			Imbricatea	Marimonadida	*Abollifer globosa* §					CPE production reported	Kashiyama et al. [6], Shiratori et al. [13]
			Thecofilosea	Cryomonadida	*Protaspa* sp.	SNK007 (A. Shihongi)	TC		(unidentified diatom) (A. Shihongi)	Detected	Unpublished strain
				Tectofilosida	*Trachyrhizium urniformis*	NIES-3876	TC		*Nitzschia* sp. (co-cultured in the original culture)	Detected	
			Paracercomonadida		*Paracercomonas* sp.§§	KMO003 (M. Kayama)	Cyano-TC		*Synechococcus elongatus* (PCC 7942), *Synechocystis* sp. (PCC 6803)	CPE production reported	Kinoshita et al. [7]
			Glissomonadida		*Orciraptor agilis*	OrcA03 (S. Hess)	TC	†	*Mougeotia* sp. (CCAC 3626)	Detected	Hess & Melkonian [14]
					Viridiraptor invadens	Virl02 (S. Hess)	TC	†	*Zygnema pseudogedeanum* (CCAC 0199)	Detected	Hess & Melkonian [14]
		Endomyxa	Vampyrellida		*Vernalophrys algivore*	(Y. Gong)	TC		*Scenedesmus acuminatus* (Y. Gong)	Detected	Gong et al. [15]
					Hyalodiscus sp.	BWH1-2 (S. Hess & K. More)	TC	†	*Tetraselmis* sp. (CCAC 6920)	Detected	Unpublished strain
					Thalassomyxa sp.	PPP (S. Hess & K. More)	TC	†	*Tetraselmis* sp. (CCAC 6920)	Detected	Unpublished strain
		Retaria	Radiolaria	Acantharia	Acnatharia spp.	(F. Not)	With algal endosymbionts		Endosymbiotic haptophyte (*Phaeocystis* sp.)	Detected	
				Polycystinea	Collodaria spp.	(F. Not)	With algal endosymbionts		*Brandtodinium nutricula* (endosymbiont)	Detected	
			Foraminifera	Globothalamea	*Ammonia* sp.	(M. Tsuchiya)	TC		*Fistulifera solaris* (T. Tanaka), *Pyramimonas parkeae* (NIES-254)	Detected	Unpublished strain
	Haptista	Haptophyta	Pavlovales		*Pavlova gyrans*	NIES-623	UC		—	N.D.	
			Prymnesiophyceae	Prymnesiales	*Chrysochromulina* sp.	NIES-1333	UC		—	N.D.	
					Prymnesium sp.	NIES-1397	UC		—	N.D.	
					Prymnesium calathiferum	NIES-1330	UC		—	N.D.	
				Isochrysidales	*Isochrysis galbana*	NIES-2590	UC		—	N.D.	
					Gephyrocapsa oceanica	NIES-353	UC	†	—	N.D.	
				Coccolithales	*Calyptrosphaera sphaeroidea*	NIES-997	UC		—	Detected	
					Ochrosphaera neapolitana	NIES-1964	UC		—	N.D.	
					Pleurochrysis roscoffensis	NIES-8	UC	†	—	N.D.	
					Umbilicosphaera foliosa	NIES-2878	UC		—	N.D.	
					Oolithotus fragilis	NIES-1320	UC		—	Detected	
				Phaeocystales	*Phaeocystis globosa*	NIES-1396	UC		—	N.D.	
		Centroplasthelida (centroheliozoans)	Pterocystida	Raphidista	*Raphidiophrys contractilis*	NIES-2498	TC	†	*Chlorogonium capillatum* (NIES-3374)	Detected	
					Choanocystis sp.	MZD010 (T. Matsuda)	TC		*Chlorogonium capillatum* (NIES-3374)	Detected	Unpublished strain
			Panacanthocystida		(unidentified)§					CPE production reported	Kashiyama et al. [6,16]
	Cryptista		Cryptophyceae	Cryptomonadales	*Chroomonas* sp.	OR1 (A. Yokoyama)	UC		—	N.D.	Unpublished strain
					Cryptmonas curvata	CCAP979/52	UC		—	N.D.	
					Cryptomonas ovata	NIES-274	UC	†	—	N.D.	
					Hemiselmis sp.	DA77 (A. Yokomama)	UC		—	N.D.	Fukuda et al. [9]
					Guillardia theta	CCMP2712	UC		—	N.D.	
					Rhodomonas sp.	DA21 (A. Yokoyama)	UC		—	N.D.	Unpublished strain
				Cyathomonadacea (goniomonads)	*Goniomonas pacifica*	NIES-1372	Cyano-TC		*Synechococcus* sp. (NIES-969)	Detected	New prey-predator relationship
					Goniomonas sp.	SNK003 (A. Shihongi)	Cyano-TC		*Synechococcus* sp. (NIES-969)	Detected	Unpublished strain
					Goniomonas sp.	SNK008 (A. Shihongi)	Cyano-TC		*Synechococcus* sp. (NIES-969)	Detected	Unpublished strain
				Kathablepharidacea	*Katablepharis japonica*	NIES-1334	TC		*Chrysochromulina* sp. (NIES-1333)	Detected	
					Leucocryptos marina	NIES-1335	TC		*Chrysochromulina* sp. (NIES-1333)	Detected	
			Palpitomonas		*Palpitomonas bilix*	NIES-2562	TC		Unidentified pedinophyte (NIES-2566)	N.D.	New prey-predator relationship, Yabuki et al. [17]
	Archaeplastida	Glaucophyta			*Glaucocystis nostochinearum*	NMR2 (M. Nomura)	UC		—	N.D.	Unpublished strain
		Chloroplastida	Chlorophyta	Pyramimonadales	*Pyramimonas parkeae*	NIES-254	UC	†	—	N.D.	
					Pyramimonas sp.	DA140 (A. Yokoyama)	UC		—	N.D.	
				Chlorodendrophceae	*Tetraselmis* sp.	(A. Yamaguchi)	UC		—	N.D.	Yamaguchi et al. [10]
					Tetraselmis sp.	CCAC 6920	UC	†	—	N.D.	
				Pedinophyceae	(unidentified)	NIES-2566	UC		—	N.D.	
				Chlorophyceae	*Chlamydomonas reinhardtii*	CC-503	UC	†	—	N.D.	
					Chlamydomonas debaryana	KMO001 (M. Kayama)	UC		—	N.D.	Unpublished strain
					Chlorogonium capillatum elongatum	(H. Dieckhoff)	UC		—	N.D.	Dieckhoff et al. [18]
					Chlorogonium capillatum	NIES-3374	UC	†	—	N.D.	
					Scenedesmus acuminatus	(C.W. Zhang)	UC		—	N.D.	Wang et al. [19]
				Trebouxiophyceae	*Chlorella* sp.	(Y. Gong)	UC		—	N.D.	Ma et al. [20]
				Ulvophyceae	*Bryopsis* sp.	(A. Yokoyama)	UC		—	N.D.	Sampled in the present study
			Streptophyta	Phragmoplastophyta	*Closterium* sp.	(Y. Kashiyama)	UC		—	N.D.	Unpublished strain
					Mougeotia sp.	CCAC 3626	UC	†	—	N.D.	Hess & Melkonian [14]
					Zygnema pseudogedeanum	CCAC 0199	UC	†	—	N.D.	
					Spinacia oleracea	(Y. Kashiyama)	UC		—	N.D.	Newly isolated strain
		Rhodophyceae (rhodophytes)	Cyanidiales		*Cyanidium caldarium*	56B (A. Yokoyama)	UC		—	N.D.	Unpublished strain
			Proteorhodophytina	Porphyridiophyceae	*Porphyridium sorididum*	AYCC163 (A. Yokoyama)	UC		—	N.D.	Unpublished strain
					Porphyridium aerugineum	AYCC558 (A. Yokoyama)	UC		—	N.D.	Unpublished strain
					Porphyridium purpureum	AYCC340/Mac01 (A. Yokoyama)	UC		—	N.D.	Takaichi et al. [21]
				Stylonematales	*Chroodactylon ornatum*	AYCC329 (A. Yokoyama)	UC		—	N.D.	Unpublished strain
					Stylonema alsidii	AYCC697 (A. Yokoyama)	UC		—	N.D.	Yokoyama & Hara [22]
					Stylonema alsidii	AYCC85 (A. Yokoyama)	UC		—	N.D.	Yokoyama & Hara [22]
			Eurhodophytina	Bangiales	*Bangia fuscopurpurea*	AYCC185 (A. Yokoyama)	UC		—	N.D.	Yokoyama & Hara [22], Takaichi et al. [21]
					Porphyra sp.	AYCC889 (A. Yokoyama)	UC		—	N.D.	Unpublished strain
				Florideophycidae	*Bostrychia moritziana*	CCAP 1357/8	UC		—	N.D.	Sekimoto et al. (2009) [23]
	Stramenopiles	Gyrista	Ochrophyta	Diatomeae	*Chaetoceros calcitrans*	HYP-C (Y Hirakawa)	UC		—	N.D.	Unpublished strain
					Skeletonema sp.	DA123 (A. Yokoyama)	UC		—	N.D.	Kashiyama et al. [6], Shiratori et al. [13]
					Skeletonema marinoi-dohrnii complex	NIES-324	UC	†	—	N.D.	
					(unidentified)	E07 (T. Shiratori)	UC		—	N.D.	Unpublished strain
					Nitzschia sp.	E06 (T. Shiratori)	UC		—	N.D.	Kashiyama et al. [6]
					Synedra sp.	S1 (M. Kagami)	UC		—	N.D.	
					Synedra sp.	TAU-kk (T. Umetani)	UC		—	N.D.	Unpublished strain
					Fistulifera solaris	(T. Tanaka)	UC		—	N.D.	Matsumoto et al. [24]
					Entomoneis sp.	E05 (T. Shiratori)	UC		—	N.D.	Kashiyama et al. [6]
						FE03 (T. Shiratori)	UC		—	N.D.	Unpublished strain
				Synchromophyceae	*Chlamydomyxa* sp.	NUS1 (A. Nakamura)	UC		—	N.D.	
				Raphidophyceae	*Heterosigma akashiwo*	DA35 (A. Yokoyama)	UC		—	N.D.	
				Chrysophyceae	*Ochromonas danica*	NIES-2142	UC	†	—	N.D.	
							TC	†	*Chlamydomonas reinhardtii* (CC-503)	N.D.	New prey-predator relationship
					Ochromonas sp.	NIES-2300	UC	†	—	N.D.	
							TC	†	*Chlamydomonas reinhardtii* (CC-503)	N.D.	New prey-predator relationship
					Poterioochromonas malhamensis	NIES-2144	UC	†	—	N.D.	
							TC	†	*Chlamydomonas reinhardtii* (CC-503)	Detected	New prey-predator relationship
					Poterioochromonas malhamensis	(Y. Gong)	UC		—	N.D.	Ma et al. [20]
							TC		*Chlorella* sp. (Y. Gong)	N.D.	
					Paraphysomonas sp.	MZD003 (T. Matsuda)	Cyano-TC		*Prochlorococcus* sp. (NIES-2882)	Detected	Unpublished strain
					Paraphysomonas vestita	NIES-1377	Cyano-TC		*Synechococcus leopoliensis* (NIES-3277)	Detected	New prey-predator relationship
					Picophagus flagellatus	NIES-2586	Cyano-TC		*Synechococcus* sp. (NIES-969)	Detected	New prey-predator relationship
				Eustigmatophyceae	(unidentified)	FE06 (T. Shiratori)	UC		—	N.D.	Unpublished strain
			Actinophryidae		*Actinophrys sol*	ATCC-50937	TC		*Chlorogonium capillatum* (NIES-3374)	Detected	New prey-predator relationship
		Gyrista	Developea		*Developayella* sp.	SRT002 (T. Shiratori)	Cyano-TC		*Synechococcus* sp. (NIES-969)	N.D.	Unpublished strain
					Developayella sp.	NIES-1388	Cyano-TC		*Synechococcus* sp. (NIES-969)	N.D.	New prey-predator relationship
					Developayella sp.	SNK010 (S. Shihongi)	Cyano-TC		*Synechococcus* sp. (NIES-969)	N.D.	Unpublished strain
			Oomycetes	Olpidiopsidales	*Olpidiopsis bostrychiae*	SEK 544 (J. West)	TC		*Bostrychia moritziana* (CCAP 1357/8)	N.D.	Sekimoto et al. [23]
		Bigyra	Sagenista	Pseudophyllomitidae	*Pseudophyllomitus* sp.§					CPE production reported	Kashiyama et al. [6]
					Pseudophyllomitus vesiculosus	SRT537 (T. Shiratori)	TC		*Chaetoceros calcitrans* (HYP-C; Y. Hirakawa)	Detected	Shiratori et al. [25]
				Labyrinthulomycetes	*Aplanochytrium kerguelnse*	KMPB N-BA-107	TC		*Skeletonema marinoi-dohrnii complex* (NIES-324)	N.D.	Honda et al. [26]
			Opalozoa	Bicosoecida	*Bicosoecida* sp.	SNK009 (A. Shihongi)	Cyano-TC		*Synechococcus* sp. (NIES-969)	Detected	Unpublished strain
					Bicosoecida sp.	SNK004 (A. Shihongi)	Cyano-TC		*Synechococcus* sp. (NIES-969)	Detected	Unpublished strain
					Cafeteria roenbergensis	NIES-1012	Cyano-TC		*Synechococcus* sp. (NIES-969)	Detected	New prey-predator relationship
					Symbiomonas scintillans	NIES-2589	Cyano-TC		*Synechococcus* sp. (NIES-969)	Detected	New prey-predator relationship
					Bicosoeca sp.	NIES-1438	Cyano-TC		*Prochlorococcus* sp. (NIES-2882)	Detected	New prey-predator relationship
				Placidida	*Placidia cafeteriopsis*	NIES-1013	Cyano-TC		*Synechococcus* sp. (NIES-969)	N.D.	New prey-predator relationship
					Wobblia lunata	NIES-1015	Cyano-TC		*Synechococcus* sp. (NIES-969)	N.D.	New prey-predator relationship
		Platysulcus			*Platysulcus tardus*	SRT153 (T. Shiratori)	Cyano-TC		*Synechococcus* sp. (NIES-969)	N.D.	Shiratori et al. [27]; new prey-predator relationship
	Alveolata	Colponemida	*Oxyrrhis*		*Oxyrrhis marina*	NIES-494	TC		*Pyramimonas parkeae* (NIES-254)	Detected	
		Dinoflagellata	Noctilucales		*Noctiluca scintillans**					CPE production reported	
			Dinophyceae	Gymnodiniphycidae	*Lepidodinium chlorophorum*	NIES-1868	UC		—	Detected	
					Amphidinium carterae	NIES-331	UC	†	—	N.D.	
					Gyrodinium falcatum	NIES-3604	UC		—	Detected	
					Karenia mikimotoi	NIES-2411	UC		—	Detected	
					Katodinium sp.	NIES-2008	UC		—	N.D.	
					Asulcocephalium miricentonis	NIES-3775	UC		—	Detected	
					*Amphidinium gibbosum**					CPE production reported	Production on phagophycophagy
					*Bispinodinium angelaceum***					CPE production reported	
					Symbiodinium sp.**					CPE production reported	
					Symbiodinium sp.**					CPE production reported	
					(unidentified)**					CPE production reported	
					(unidentified)**					CPE production reported	
					Symbiodinium sp.***					CPE production reported	
				Peridiniphycidae	*Alexandrium catenella*	NIES-675	UC	†	—	Detected	
					Coolia monotis	NIES-343	UC	†	—	N.D.	
					Glenodiniopsis uliginosa	NIES-463	UC	†	—	Detected	
					Heterocapsa niei	NIES-420	UC	†	—	Detected	
				Prorocentrales	*Prorocentrum micans*	NIES-12	UC	†	—	N.D.	
		Ciliophora	Oligohymenophorea	Peniculia	*Frontonia* sp.	(Y. Gong)	Cyano-TC		*Arthrospira* sp. (Y. gong)	Detected	Unpublished strain
					Paramecium bursaria	(T. Suzaki)	With algal endosymbionts		Endosymbiotic *Zoochlorella* sp.	N.D.	Unpublished strain
					Paramecium caudatum	(T. Suzaki)	TC		*Chlorogonium capillatum* (NIES-3374)	N.D.	Unpublished strain
					Paramecium multinucleatum	(T. Suzaki)	TC		*Chlorogonium capillatum* (NIES-3374)	N.D.	Unpublished strain
			Spirotrichea	Euplotia	*Euplotes woodruffi*	(R. Higuchi)	TC		*Chlorogonium capillatum* (NIES-3374)	N.D.	Unpublished strain
				Perilemmaphora	*Strombidinopsis acuminatum**					CPE production reported	
					Sterkiella histriomuscorum	(Y. Gong)	TC		*Chlorogonium capillatum* (Y. Gong)	N.D.	Unpublished strain
	(incertae sedis)		Telonemia		*Telonema* sp.	(A. Yabuki)	TC		Unidentified pedinophyte (NIES-2566)	Detected	Yabuki et al. [28]
		Discosea	Centramoebia	Acanthopodida	*Acanthamoeba* sp.	(A. Uzuka)	TC		(unidentified green alga; A. Uzuka)	N.D.	Unpublished strain
			Flabellinia	Dermamoebida	*Mayorella* sp.	TAU001 (T. Umetani)	TC		*Synedra* sp. (TAU-kk; T. Umetani)	N.D.	Unpublished strain
					Mayorella sp.	(T. Suzaki)	TC		*Chlorogonium capillatum* (NIES-3374)	N.D.	Unpublished strain
				Dactylopodida	(unidentified)	(A. Uzuka)	TC		(unidentified green alga; A. Uzuka)	N.D.	Unpublished strain
					Neoparamoeba sp.	EVE002 (J. Taira, *et al*.)	TC		*Fistulifera solaris* (T. Tanaka)	Detected	Unpublished strain
		Tubulinea	Elardia	Arcellinida	(unidentified)	SRT201 (T. Shiratori)	TC		(unidentified diatom) (FE03; T. Shiratori)	Detected	Unpublished strain
		Evosea			(undescribed genus)	(S. Hess)	TC	†	*Zygnema pseudogedeanum* (CCAC 0199)	Detected	Unpublished strain
	Opisthokonta	Nucletmycea	Rotosphaerida		(unidentified)	SRT113 (T. Shiratori)	TC		*Entomoneis* sp. (E05; T. Shiratori)	N.D.	Kashiyama et al. [6]
					Pinaciophora sp.	SRT227 (T. Shiratori)	TC		(unidentified Thalassiosirales) (E07; T. Shiratori)	N.D.	Unpublished strain
			Chytridiomycota	Chytridiales	*Rhizophydium planktonicum*	AstB5 (M. Kagami)	TC		*Asterionella formosa* (M. Kagami)	Detected	
					Zygorhizidium melosirae	KS99 (K. Seto)	TC		*Aulacoseira granulata* (K. Seto)	Detected	Seto et al. [29]
					Zygorhizidium melosirae	C1 (K. Seto)	TC		*Aulacoseira ambigua* (K. Seto)	N.D.	Seto et al. [29]
				Rhizophydiales	(unidentified)	E1 (M. Kagami)	TC		*Synedra* sp. (S1; M. Kagami)	Detected	
				(unidentified)		C3 (M. Kagami)	TC		*Aulacoseira ambigua* (M. Kagami)	N.D.	
	Metamonada			Fornicata	*Dysnectes brevis*	NIES-1843	Cyano-TC		*Synechococcus* sp. (NIES-969)	N.D.	New prey-predator relationship
	(incertae sedis)			Collodictyonidae	*Diphylleia rotans*	NIES-3764	Cyano-TC		*Microcystis aeruginosa* (NIES-298)	N.D.	New prey-predator relationship
Cyanobacteria					*Arthrospira platensis*	(Y. Gong)	UC		—	N.D.	Unpublished strain
					Microcystis aeruginosa	NIES-298	UC		—	N.D.	
					Prochlorococcus marinus	NIES-2087	UC		—	N.D.	
					Prochlorococcus sp.	NIES-2882	UC		—	N.D.	
					Synechococcus elongatus	PCC 7942	UC		—	N.D.	
					Synechococcus leopoliensis	NIES-3277	UC		—	N.D.	
					Synechococcus sp.	NIES-969	UC		—	N.D.	
					Synechocystis sp.	PCC 6803	UC		—	N.D.	
